# Transient pores in hemifusion diaphragms

**DOI:** 10.1016/j.bpj.2024.06.009

**Published:** 2024-06-11

**Authors:** Russell K.W. Spencer, Yuliya G. Smirnova, Alireza Soleimani, Marcus Müller

**Affiliations:** 1 Institute for Theoretical Physics, Georg-August University, Göttingen, Germany; 2 Technische Universität Dortmund, Dortmund, Germany

## Abstract

Exchange of material across two membranes, as in the case of synaptic neurotransmitter release from a vesicle, involves the formation and poration of a hemifusion diaphragm (HD). The nontrivial geometry of the HD leads to environment-dependent control, regarding the stability and dynamics of the pores required for this kind of exocytosis. This work combines particle simulations, field-based calculations, and phenomenological modeling to explore the factors influencing the stability, dynamics, and possible control mechanisms of pores in HDs. We find that pores preferentially form at the HD rim, and that their stability is sensitive to a number of factors, including the three line tensions, membrane tension, HD size, and the ability of lipids to “flip-flop” across leaflets. Along with a detailed analysis of these factors, we discuss ways that vesicles or cells may use them to open and close pores and thereby quickly and efficiently transport material.

## Significance

This study explores the complex dynamics and stability of pores in hemifusion diaphragms (HDs), critical intermediates to fusion processes like synaptic neurotransmitter release. By integrating particle simulations, field-based calculations, and phenomenological modeling, we explore the factors influencing pore formation and stability in HDs. Our findings illuminate the preferential formation of pores at the HD rim and their sensitivity to line tensions, membrane tension, HD size, and lipid dynamics. This research not only advances our understanding of the HD behavior but also sheds light on potential cellular mechanisms for controlling pore dynamics, offering significant implications for the broader fields of neurobiophysics and membrane biophysics.

## Introduction

Neurotransmitter release is a critical process governing synaptic communication. Small synaptic vesicles (SVs) fuse and release neurotransmitters (exocytosis) at the active zone of the presynapse, and are subsequently retrieved by endocytosis. The morphological uniformity and protein composition of SVs is maintained over repetitive rounds of exo- and endocytosis to sustain neurotransmission. Thus, it is expected that the exocytotic vesicle fusion in the active zone and the endocytotic retrieval of SV membranes are tightly coupled in time and space. The precise mechanisms of synaptic release have garnered significant interest in the realm of neurobiophysics (1,2,3,4,5,6,7).

Two principal mechanisms have been postulated for this release: 1) the full fusion of vesicles with the presynaptic membrane in conjunction with subsequent, clathrin-mediated endocytosis (8), and 2) the “kiss-and-run” (K&R) mechanism (9,10,11,12,13,14). In the full-fusion mechanism, SVs completely merge with the synaptic membrane, releasing their contents into the synaptic cleft. In contrast, the K&R mechanism posits a transient fusion, wherein a vesicle fuses, forms a pore to release some neurotransmitters, and then disconnects without undergoing full fusion and collapse into the presynaptic membrane (1,10,11). Evidence suggests that vesicles may fuse transiently in succession without losing their identity (15,16).

**Figure 1:**
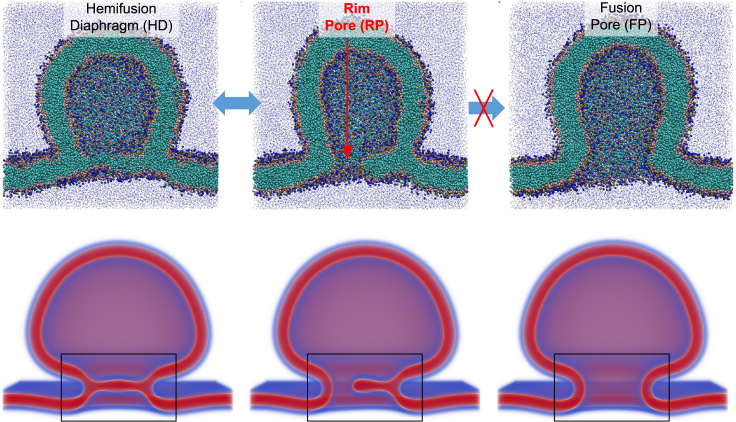
Qualitative illustration of the fusion of a vesicle with a membrane, as described using particle-based simulations of the coarse-grained MARTINI model (*upper row*) and self-consistent field theory (SCFT) (*lower row*). The vesicle starts locally fused with the membrane, by way of a hemifusion diaphragm (HD). A pore then forms at the rim of the HD and expands to form a fusion pore. This work focuses on the HD itself, which is highlighted in a box. To see this figure in color, go online.

Central to our understanding of the K&R mechanism is the role of the hemifusion diaphragm (HD), an intermediate structure formed as vesicles fuse with the presynaptic membrane. A sketch of such a fusion process is shown in Fig. 1. Within this context, the stability and dynamics of transient or “flickering” pores within the HD are of paramount importance, as they serve as the conduit for neurotransmitter release (12,17,18,19). Theoretical and experimental insights into the HD behavior suggest that its dynamics, especially pore formation and expansion, present significant free-energy barriers, emphasizing the need for rigorous control of the HD size for fine-tuning fusion dynamics (20,21,22,23,24,25). This work employs a combination of theoretical approaches to study transient pores in HDs as a function of the HD’s environment, specified by chemical or mechanical constraints.

Poration has been extensively studied and is relatively well understood (26,27,28,29,30). However, the introduction of the HD leads to a variety of complexities: 1) Rim pores (formed at the edge of the HD) (31,32,33,34), for example, give rise to an interplay between the shape of the rim pore and the HD, owing to the multiple line and membrane tensions involved. 2) Moreover, the ability for HDs to change size makes the region outside of the HD effectively a lipid reservoir, further complicating classical descriptions of pores. Elucidating the stability and dynamics of rim pores within HDs demands considering the composite shape of rim pore and HD, and its coupling to the environment.

The landscape of theoretical methodologies offers a variety of tools. On the fine-scaled end of the spectrum, there are particle-based approaches (23,35,36,37,38,39,40,41,42,43,44,45). These offer a precise method for modeling the behavior of lipids and thus the membranes that emerge from them. Rather than all-atom simulations, we employ the coarse-grained MARTINI model (46), which captures the behavior of the lipids (47) without the computational cost of atomistic approaches. Even though the MARTINI model is coarse grained, and thus fast, on the scale of particle simulations, it can still be inefficient to study large-scale membrane rearrangements, observe rare events, or explore large, multidimensional parameter spaces.

On the other resolution extreme, there are phenomenological free-energy approaches, such as Helfrich models (24,48,49,50,51,52,53,54). The basic object in these calculations is the membrane itself, rather than the molecules that compose it. The membrane is conceived as a sheet-like object, and its free-energy functional is written in terms of the membrane shape, using properties, such as membrane areas, bending energies, line tensions, etc., as given phenomenological parameters. This far more coarse-grained class of approaches can often offer greater physical insight than particle simulations, using orders of magnitude less computational resources. Their accuracy, however, relies on the accuracy of the phenomenological parameters and, more importantly, the accuracy of the free-energy functional itself, leading to difficulties with, for example, highly curved configurations, since the bending energy begins to depend on the curvature (55). Further difficulties arise when nonsheet-like membrane structures, such as worm-like micelles or stalks, changes in topology, such as pore formation, and other nontrivial membrane shapes need to be considered. Fortunately, our particular problem contains mostly planar membranes.

At an intermediate level of coarse graining, there is self-consistent field theory (SCFT) (56,57). This approach has been used for a variety of polymer and membrane applications (58,59,60,61,62,63,64,65,66). It works by calculating the statistics of lipids in an ensemble, and thus studies the membrane as emergent from lipid statistics, without having to track each individual molecule. It is faster and more adaptable than particle-based simulations, and allows us to easily change lipid types and interactions, enabling us to swiftly explore parameter space, while still having the membrane emerge from lipid statistics and thus avoiding the shortcomings of a Helfrich-like description. SCFT is primarily used for equilibrium calculations, but can be modified to examine transformations of membrane shapes, including changes of membrane topology. The string method (41,42) gives us a way to find the optimal path—the minimum free-energy path (MFEP)—connecting stable or metastable states. This is done without having to impose a reaction coordinate, as one is derived in terms of local changes in lipid concentration.

This work combines the three approaches described above: first, we derive a phenomenological model for rim pores to understand their behavior in terms of phenomenological membrane parameters. The next step is to use molecular dynamics simulations of the MARTINI model and SCFT calculations to obtain the phenomenological parameters and test the predictions of the phenomenological model. This allows us to make precise predictions regarding the formation and stability of rim pores in HDs, and provides insights into how cellular systems may control these pores during synaptic neurotransmitter release.

## Models and methods

### Phenomenological model of a rim pore

**Figure 2:**
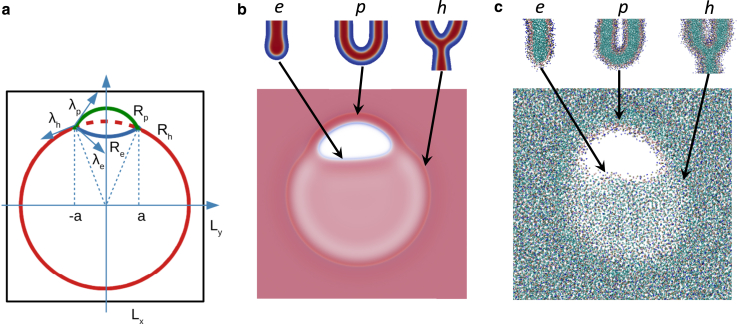
Rim pores are shown (*a*) as described in the phenomenological model and in (*b*) SCFT and (*c*) particle-based simulations with depictions of each type of bounds: pore edge, *e*, fusion pore, *p*, and three-bilayer junction, *h*. The sketch, (*a*), shows a HD, with radius *R*_*h*_ (outlined in *red*), which has a rim pore (RP) of width 2a. The outer (*green*) and inner (*blue*) interfaces of the RP have radii of curvature $R_{p}$ and $R_{e}$, respectively. Line tensions, labeled λ exert forces along the blue arrows. The dimensions of the system along *x* and *y* axes are denoted as $L_{x}$ and $L_{y}$, respectively. The widths of the SCFT and particle-based simulations are ≈60 and ≈45nm, respectively. To see this figure in color, go online.

Describing the rim pore in an HD (see Fig. 2) in terms of a minimal set of intuitively identifiable parameters, we obtain insights into its stability in an HD, which we then use to guide and interpret the findings of our other approaches. Our minimal, phenomenological model is sketched here, and we provide a more complete derivation in Appendix A: phenomenological model.

Our general approach is as follows: the fused membranes have less total area than a simple pair of apposing membranes, owing to a double-membrane region being replaced with a single membrane, the HD. We characterize the system based on this difference in area, $A_{h0}$, and the distance, 2d, between the centers of mass of the two apposing membranes. In addition, the geometry of the rim pore is characterized by the three radii—$R_{h}$ of the HD, $R_{e}$ of the edge of the pore in the HD, and $R_{p}$ of the fusion-pore-like edge of the pore—and the pore half-width, *a*, as depicted in Fig. 2. The molecular structure of the three line segments of the rim pore is also presented in Fig. 2, obtained from models that account for the lipid architecture. In the minimal phenomenological model, we also incorporate their line tensions, $\lambda_{h}$, $\lambda_{e}$, and $\lambda_{p}$, that quantify the excess free energy per unit length.

Given this area, $A_{h0}$, and the distance, *d*, of the apposing membranes together with the line tensions, $\lambda_{h}$, $\lambda_{e}$, and $\lambda_{p}$, described in Fig. 2, we write the free energy as a function of the geometry of the rim pore in the HD, i.e., in terms of $R_{h}$, $R_{e}$, and $R_{p}$. The parameter *a* is dictated by the constraint of $A_{h0}$. We optimize this free energy at fixed membrane area, producing geometries where each point, most notably the vertices of the pore, have no net force. We subsequently discuss the optimal rim pore geometry and its stability, studying the curvature of the free energy in the $R_{h}-R_{e}-R_{p}$ space. In the limiting case d=0, the HD area is equal to $A_{h0}$; however, for finite *d*, some membrane area goes to connecting membranes together, leading to a smaller HD area. We focus on the d=0 case, as it contains the important aspects of the model; however, we also present the finite *d* corrections. Where applicable, we include the dependence on *d* in $A_{h0}\left(d\right)$, which is derived in the appendix.

### SCFT

SCFT gives us a method for describing the mesoscale behavior of lipid membranes by way of calculating the statistics of lipids (56,57). Membranes self-assemble in a system of volume *V* containing $n_{l}$ lipids and $n_{s}$ water molecules, modeled as AB diblock copolymers and short homopolymers, respectively. The molecular statistics are calculated using the Gaussian chain model, with lipids having *N* statistical segments, of length *b*, and of which fN are hydrophobic tails and (1−f)N are heads. The effective repulsion between heads and tails or water and tails is characterized by the Flory-Huggins parameter, χN. The water molecules are smaller, having N/10 segments.

Starting from an initial membrane configuration (lipid concentration profile), we can calculate the potentials felt by the lipids and thereby also calculate the statistics of the lipids and thus their concentration profile. This is in general different from the starting configuration. The difference gives us local exchange chemical potentials that we use to update the profile until it is self-consistent. Alternatively, we can construct a path through configuration space, and optimize it, using the local exchange chemical potentials, to obtain a MFEP. This is the string method. Our approach closely mirrors that used in Ref. (67) and a detailed description of SCFT and the string method is given in Appendix B: Self-consistent field theory implementation.

The natural length scale in SCFT is the average end-to-end length of the lipid, $R_{0}=b\sqrt{N}$. To map our SCFT results onto simulations or experiments, we use the membrane thickness, which is $D\approx 1.2R_{0}$ in our SCFT results and D≈4nm in experiments (68). The free energy in SCFT is scaled by $\sqrt{\bar{N}}k_{B}T$, where $\bar{N}$ is the invariant polymerization index, and varies with the length of the lipid and density of the system. We also calculate the bilayer bending modulus, which is $\kappa =0.209\sqrt{\bar{N}}k_{B}T$ for our typical SCFT parameter choice of χN=30 and f=0.8. This allows us to express free energies in units of *κ*.

SCFT is conducted at an intermediate level of coarse graining, between the phenomenological approach and particle-based simulations. Calculating the behavior of membranes from the statistical behavior of the lipids does not require us to impose properties like bending energies, etc., as these emerge from the underlying lipid behavior, while being less computationally costly than particle-based simulations, as we do not need to consider each individual particle, but rather calculate the statistics of each molecular species as a whole.

### Coarse-grained particle simulation

In the coarse-grained simulations, the MARTINI model was employed to represent water and lipid molecules (46). We simulated DMPC (1,2-ditetradecanoyl-*sn*-glycero-3-phosphocholine with double saturated tails) lipids, with 10 coarse-grained beads in total. For a few cases we additionally studied POPC (1-palmitoyl-2-oleoyl-glycero-3-phosphocholine with a saturated chain in the *sn*-1 position and an unsaturated chain in the *sn*-2 position) lipids, which have 13 coarse-grained beads in total. The difference of these two lipids is in the tail length, whereas the headgroups are the same. DMPC is characterized by a positive spontaneous monolayer curvature, (0.135±0.002) nm^−1^ (69), whereas POPC has a small negative spontaneous curvature, (−0.022±0.010) nm^−1^ (70), due to its long, unsaturated acyl chain. The molecular dynamics simulations were performed with GROMACS (71).

### Calculating line tensions

**Figure 3:**
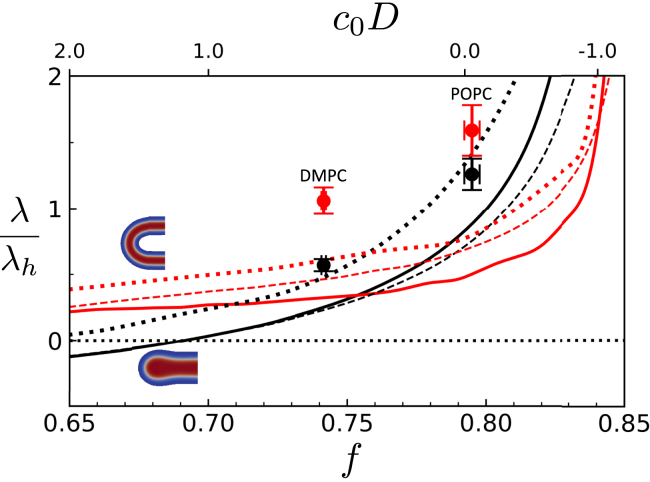
Line tensions from SCFT (*lines*) and particle-based simulations (*points*). Data correspond to dimensionless ratios, $\lambda_{p}/\lambda_{h}$ (*red*) and $\lambda_{e}/\lambda_{h}$ (*black*). Error bars indicate uncertainties in the spontaneous curvature and line tension from MARTINI calculations. Line tensions from SCFT are shown as functions of the volume fraction of the hydrophobic tail, *f*, or the spontaneous monolayer curvature, $c_{0}$, in units of the membrane thickness, D≈4 nm (*alternate abscissa*), and are calculated for χN=30 and 2d≈2.75D≈11nm (*solid*), χN=30 and 2d≈1.75F≈7nm (*dashed*), and χN=45 and 2d≈2.75D≈11nm (*dotted*). The majority of this work uses DMPC lipids in particle-based simulations and f=0.8 and χN=30 for SCFT, where the preferred spacing near HDs is typically 2d≈2.75D≈11nm. Note that $\lambda_{p}\approx \pi \kappa /2d$, thus the corresponding value is $\lambda_{p}/\lambda_{h}\approx \pi \kappa /2d\lambda_{h}$. Absolute line tensions are shown in Fig. 15. To see this figure in color, go online.

Using SCFT and the coarse-grained particle simulation, we measure the line tensions for the three relevant system configurations, depicted in Fig. 2, via the pressure anisotropy (72) (see Eq. 29 in Appendix C: Molecular dynamics simulation and techniques). The results for the ratio of the line tensions are presented in Fig. 3 for the two MARTINI lipids and different lipid architectures in SCFT. The data are compared using the dimensionless product of monolayer spontaneous curvature, $c_{0}$, and membrane thickness, *D*.

Fig. 3 demonstrates the influence of the molecular structure and bilayer distance, *d*, on the materials parameters, $\lambda_{h}$, $\lambda_{p}$, and $\lambda_{e}$ of the phenomenological model of a rim pore. In our model, we can scale out one of the line tensions, without loss of generality (see Appendix A: Phenomenological model). We normalize by $\lambda_{h}$, as it characterizes the HD itself, and also because this scaling better emphasizes the regions in line tension space that are of interest to us (vide infra). $\lambda_{h}$ decreases as the tail size, *f*, increases. This is expected since the inverted hexagonal phase becomes stable in block copolymers. $\lambda_{p}$ is controlled by the bending modulus, κ, and the bilayer separation, *d*, and is relatively insensitive to head-tail asymmetry. $\lambda_{e}$ increases with *f*, as the edge is stabilized by large headgroups. $\lambda_{e}/\lambda_{h}$ thus increases more quickly than $\lambda_{p}/\lambda_{h}$. When the interaction parameter, χN, is increased, all three line tensions increase, although at different rates. The number of unfavorable contacts increases with curvature of the head-tail interface, providing a significant contribution to each of the line tensions.

## Results

To understand the formation, stability, and control of pores in HDs, we develop a phenomenological model, in terms of the line tensions of the distinct parts of the rim pore, and thereby predict a phase diagram of pore stability. This tells us when rim pores are stable and also provides insights into how cellular systems can control these rim pores. We turn to our less coarse-grained models, SCFT and molecular dynamics simulation, to investigate the relevant regions of the phase diagram, test our predictions, and get further insights into pore stability.

### Predicting rim pore stability

**Figure 4:**
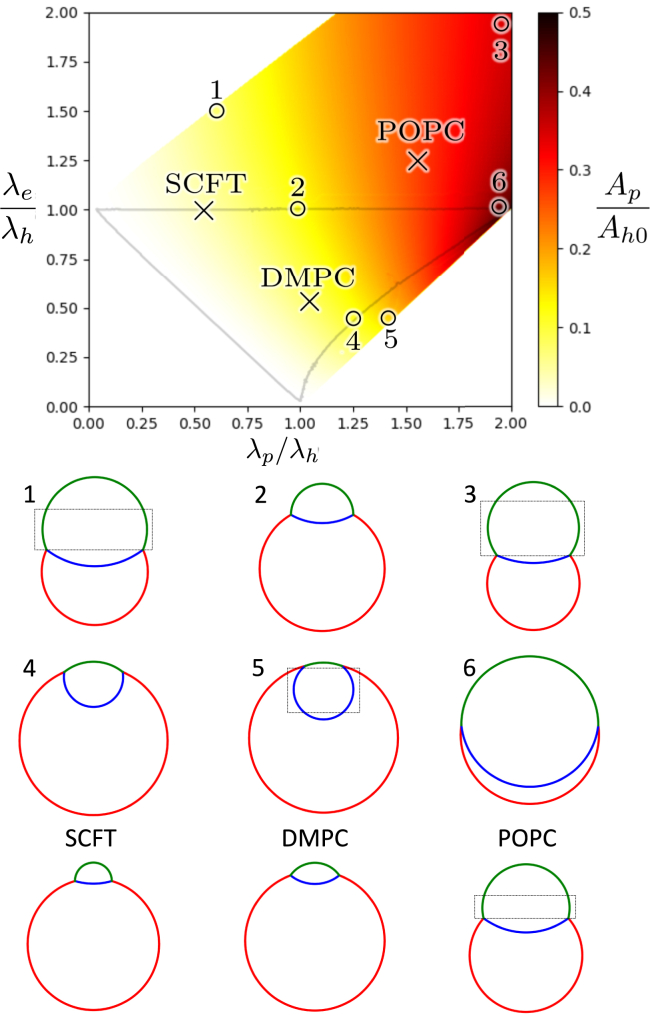
Critical rim pore area fraction as a function of the two line-tension ratios, $\lambda_{e}/\lambda_{h}$ and $\lambda_{p}/\lambda_{h}$. Critical pore geometries are shown for our simulated systems and key points to illustrate the nature of instabilities. These data are calculated from the phenomenological model as saddlepoints, $dF/dR_{e}=dF/dR_{p}=dF/dR_{h}=0$, with *a* chosen to satisfy the area constraint. The membrane separation is negligible, d=0. Data are absent where no saddlepoints exists. The gray line denotes where the pore goes from recessed to protruding either inward or outward. The extra “protruding” portions (see Λ terms discussed in the appendix) are highlighted by a rectangular box in illustrations 1, 3, and 5, and POPC. To see this figure in color, go online.

The phenomenological model, described above and in the appendix, allows us to explore the geometry and stability of rim pores, at various line-tension combinations. These correspond to different lipid architectures and other physical parameters, as illustrated in Fig. 3. The fractional rim pore area, $A_{p}/A_{h0}$, as a function of the dimensionless ratios of line tensions, $\lambda_{e}/\lambda_{h}$ and $\lambda_{p}/\lambda_{h}$, is presented in Fig. 4, only for line-tension combinations at which critical rim pores exist, i.e., the free energy exhibits a saddlepoint as a function of $R_{h}$, $R_{e}$, and $R_{p}$, corresponding to pores lacking any forces acting to open or close them, making them simpler to stabilize. The pore’s chord length along the HD rim, *a*, and diaphragm radius, $R_{h}$, are coupled by fixing the total membrane area. In the regions missing from the plot, the free energy cannot be optimized. The reasons for this are discussed briefly later. Relevant geometries of the rim pore in the HD are also illustrated in Fig. 4.

In our phenomenological model, however, rim pores are always unstable, i.e., the lowest eigenvalue of the Hessian matrix of the free energy is always negative. This is in marked contrast to simple pores in a membrane that are stable at fixed membrane area (73). To understand this lack of stability more clearly, we examine the dependence of the free energy on the pore geometry, and find that the unstable mode (eigenvector of the Hessian) has a large projection onto the radius, $R_{h}$, of the HD, i.e., the free energy is concave with respect to $R_{h}$ and convex with respect to $R_{e}$ and $R_{p}$. Thus, a small decrease of $R_{h}$ with respect to the saddlepoint value results in a runaway shrinkage of the rim pore. A minuscule growth perturbation of $R_{h}$, in turn, leads to the opposite. The saddlepoint corresponds to a critical rim pore. As with a simple pore in a single membrane *at constant membrane tension*, Fig. 4 demonstrates that the critical rim pore area increases with line tension but the relationship is complicated by 1) the presence of multiple line tensions and 2) the fact that the membrane tension is not controlled but is related to the size of the rim pore.

The observation that the unstable mode corresponds to the variation of $R_{h}$, however, suggests that mechanically restraining changes in $R_{h}$ may stabilize the pore. To test this possibility, we simulate fixing $R_{h}$ by excluding it from the Hessian matrix calculation, resulting in a positive lowest eigenvalue. Fixing $R_{h}$ can thus stabilize the geometry, converting critical pores into metastable ones.

**Figure 5:**
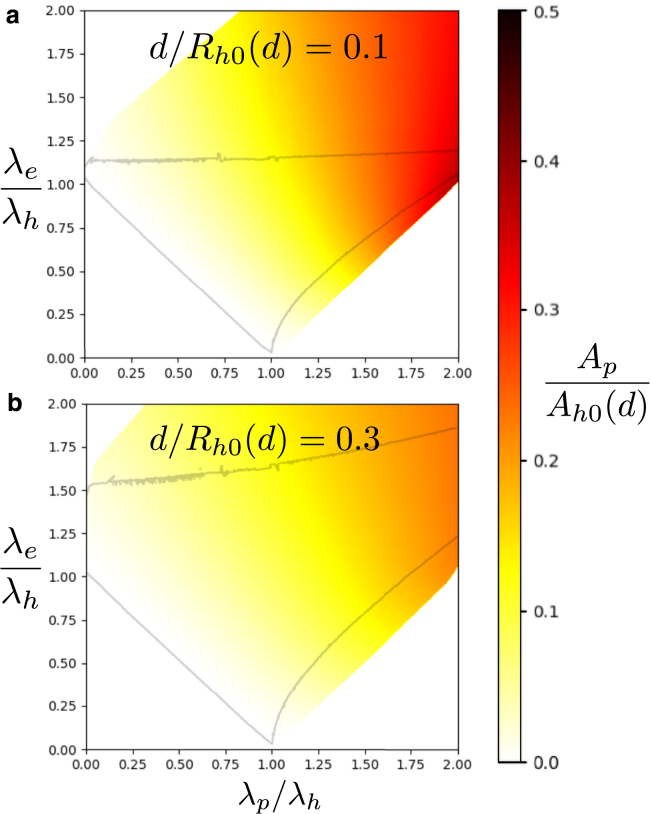
Critical rim pore area fractions similar to those shown in Fig. 4, but accounting for the finite separation, 2d, between the apposing membranes. Data are shown for (*a*) $d/R_{h0}\left(d\right)=0.1$ and (*b*) $d/R_{h0}\left(d\right)=0.3$, where $R_{h0}\left(d\right)$ is the radius of an HD without rim pore. The outlined region is shifted and expanded compared with Fig. 4. Note that the reference HD area, $A_{h0}\left(d\right)$, increases with *d* as described in the appendix. To see this figure in color, go online.

For small $\lambda_{e}/\lambda_{h}$ and $\lambda_{p}/\lambda_{h}$, i.e., the bottom left quadrant in Figs. 4 and 5, we notice that the fractional area of the critical rim pore tends to 0 as we approach $\lambda_{e}+\lambda_{p}=\lambda_{h}$. To understand this instability, consider a small rim pore. If $a\ll R_{e}$ and $R_{p}$, rim pore growth can be thought of as a simple expansion along the rim of the HD, i.e., converting a line of hemifusion-like junction into a fusion-pore-like line on one side and a membrane-edge-like line on the other. If $\lambda_{e}+\lambda_{p}<\lambda_{h}$, this unzipping of the HD lowers the free energy and the rim pore will grow, i.e., even the smallest rim pores are supercritical and will expand into fusion pores. This simple explanation ignores the curvature of the HD rim; however, curvature would lead to the production of less membrane edge, lowering the free energy and enhancing the instability.

Moving upward, when $\lambda_{e}=\lambda_{h}$, the corresponding radii of curvature are also equal, $R_{e}=R_{h}$, because of the Laplace relation between line tension, curvature radius, and membrane tension. We thus see that the horizontal components of the tension, pulling on the junction point, from these two interfaces are equal in magnitude. The *p*-type interface, aka the fusion-pore-like section of the rim pore, must thus only have a vertical contribution, i.e., produce a semicircular arc, thus $R_{p}=a$. The line $\lambda_{e}=\lambda_{h}$ thus denotes the limit where the *p*-type interface begins to bulge outward, as we see in Fig. 4. This changes the nature of the instability, as a protruding rim pore may be “pinched off” to separate it from the HD, and bud off a fusion pore. At the line $\lambda_{e}=\lambda_{p}+\lambda_{h}$, the edge tension is too strong and cannot be balanced by the other two.

We next turn our attention to the bottom right (small $\lambda_{e}$, large $\lambda_{p}$). To understand the instability consider the free-energy change associated with detaching a pore from the rim, and bringing it into the center of the HD. This change replaces the *p*-type interface with an *h*- and an *e*-type and is preferable when $\lambda_{e}+\lambda_{h}<\lambda_{p}$. The adjacent gray curve denotes when the edge is semicircular. Using the Laplace relations, $R_{e}=\lambda_{e}/\Sigma$, $R_{h}=\lambda_{h}/\Sigma$ and $R_{p}=\lambda_{p}/2\Sigma$ and setting the horizontal components of the tension from the *p*-type and *h*-type interfaces equal, we find the relationship $\lambda_{e}=\sqrt{\left(\lambda_{p}^{2}-\lambda_{h}^{2}\right)/3}$, which is consistent with the separation found numerically from the phenomenological model.

The area constraint employed in the calculations above assumes that the membrane area used to vertically connect the apposing membranes along the *p*-type and *h*-type interfaces is negligible. This approximation is only accurate for very large HDs, where the membrane separation, 2d, is much smaller than the other length scales; otherwise changing the structure of the different types of lines changes the amount of membrane area available for planar membranes. Accounting for this effect is detailed in the appendix, and the resulting plots of fractional rim pore area are shown in Fig. 5. The augmented phenomenological model extends the region where a saddlepoint of the free energy exists in the $\lambda_{e}/\lambda_{h}-\lambda_{p}/\lambda_{h}$ plane and adjusts the region where pores are recessed. Specifically, larger *d* allows critical rim pores to exist at larger $\lambda_{e}/\lambda_{h}$. At fixed values of line-tension ratios, the fraction of the critical rim pore decreases with *d*.

### Testing rim pore stability

**Figure 6:**
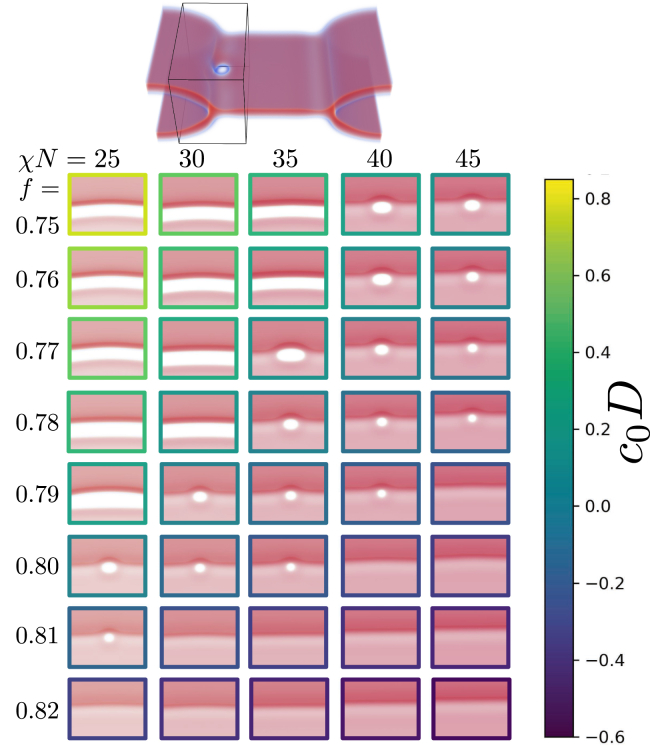
Rim pores under zero tension (linear three-bilayer junction) for different lipid architectures (tail-volume fraction, *f*) and interaction strengths, χN. Each image was extracted from a system like the one shown at the top, seen from above. For small *f* the line tensions are such that the system can reduce its free energy by growing a rim pore, converting the three-bilayer junction (*h*-type interface with line tension, $\lambda_{h}$) into a segment of a fusion pore, $\lambda_{p}$, and a membrane pore/edge, $\lambda_{e}$. For larger *f* this is no longer true, and the pore shrinks. The headgroup repulsion, however, prevents the rim pore from shrinking away entirely, making it metastable. For even larger *f*, the lipid head is not large enough to hold the pore open and it closes. There is thus a small band, in the χN−f plane, where the rim pore is metastable at zero tension. The spontaneous monolayer curvature, $c_{0}$, is shown in units of the membrane thickness, *D*. A more detailed plot is shown in Fig. 16. To see this figure in color, go online.

We first examine the stability of small rim pores in SCFT by inserting pores into linear diaphragms i.e., three-bilayer junctions. The linear junction produces a tensionless membrane: if there were tension in the membrane, then the two membranes would exert twice the tension on the *h*-type interface as one membrane. This force imbalance shifts the three-bilayer junctions until the membranes are tensionless. After insertion, pores are relaxed via the SCFT algorithm, and the corresponding results are shown in Fig. 6. We control the line tensions by changing the head-tail repulsion strength, χN, and tail-volume fraction, *f*. Pores either grow or shrink, depending on the sign of $\lambda_{p}+\lambda_{e}-\lambda_{h}$. For small χN and *f* (*top left* of Fig. 6), $\lambda_{p}+\lambda_{e}<\lambda_{h}$ and the rim pore grows. For large χN and *f* (*bottom right* of Fig. 6), in turn, $\lambda_{p}+\lambda_{e}>\lambda_{h}$, and the pore shrinks.

In addition to these two behaviors, we observe a diagonal stripe in the χN−f plane, where the rim pore neither expands nor shrinks all the way. The tendency to shrink is small, as $\lambda_{p}+\lambda_{e}≳\lambda_{h}$, and it appears that the repulsion of overlapping headgroups is sufficient to keep the rim pore open. We denote these structures as pre-rim pores, in analogy to small, metastable pores in single membranes (74,75,76). Under similar conditions, the head repulsion is not sufficient to keep a pre-pore open in a single membrane (74,75,76) but we speculate that the weak tendency to shrink combined with the smaller membrane curvature, and thus higher headgroup density, at the fusion-pore-like line segment is sufficient to keep these pre-pores open at the HD rim.

**Figure 7:**
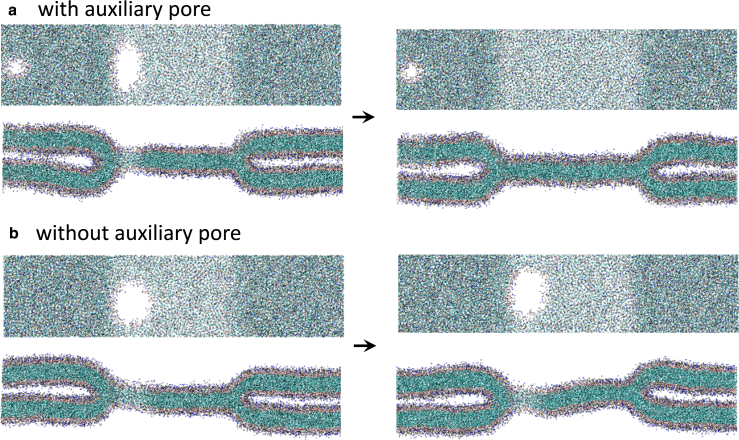
Systems similar to Fig. 6 in molecular dynamics simulations of DMPC lipids. Rim pores are introduced and allowed to stabilize either (*a*) with or (*b*) without an auxiliary pore, which allows lipid exchange between the *cis* and *trans* leaflets. The presence of the auxiliary pore (*a*, left boundary) dictates the stability of the rim pore. To see this figure in color, go online.

Similar systems, examined by molecular dynamics simulations, are shown in Fig. 7. Unlike SCFT, the barrier to lipid flip-flops between the two leaflets of a bilayer membrane is high. To facilitate flip-flops we run these simulations with two auxiliary pores, connecting the three distinct leaflets. If we allow flip-flops, lipids can move freely across the membrane, as in the SCFT calculations, and the position of the three-bilayer junction can simply move until there is zero tension in the membrane. As the line tensions satisfy $\lambda_{e}+\lambda_{p}>\lambda_{h}$ (see Fig. 3), the rim pore does not expand to unzip the three-bilayer junction. Instead, it shrinks and closes completely as shown in the top row of Fig. 7.

If flip-flops are not enhanced in the molecular dynamics simulations, the area of each leaflet is effectively conserved and leaflets behave independently. The inner *cis* leaflet, i.e., the monolayers that are closest to one another in the apposing membranes and are not connected to the HD, may contract and thereby alter $R_{h}$ until tensionless. The outer *trans* leaflets that form the HD, however, cannot change area and so may have a finite tension. Since the rim pore is, topologically, a structure in the outer *trans* leaflets, it behaves like a membrane pore in the canonical NVT ensemble. To shrink, the rim pore increases the *trans* leaflets’ area and, in turn, the membrane tension inside the HD. The rim pore radius therefore only shrinks until the Laplace tension of the curve edge of the rim pore balances the membrane tension in the HD. This balance is complicated as the line tension is a combination of $\lambda_{e}$ and $\lambda_{p}$, but the situations remain qualitatively analogous to a pore in a single membrane (73).

The majority of our molecular dynamics simulations study DMPC lipids, because their membrane properties give rise to reasonably sized, nonprotruding critical pores. In contrast, the line tensions for POPC membranes produce critical pores that protrude from the HD, deforming it into a dumbbell shape, as shown in Fig. 4.

**Figure 8:**
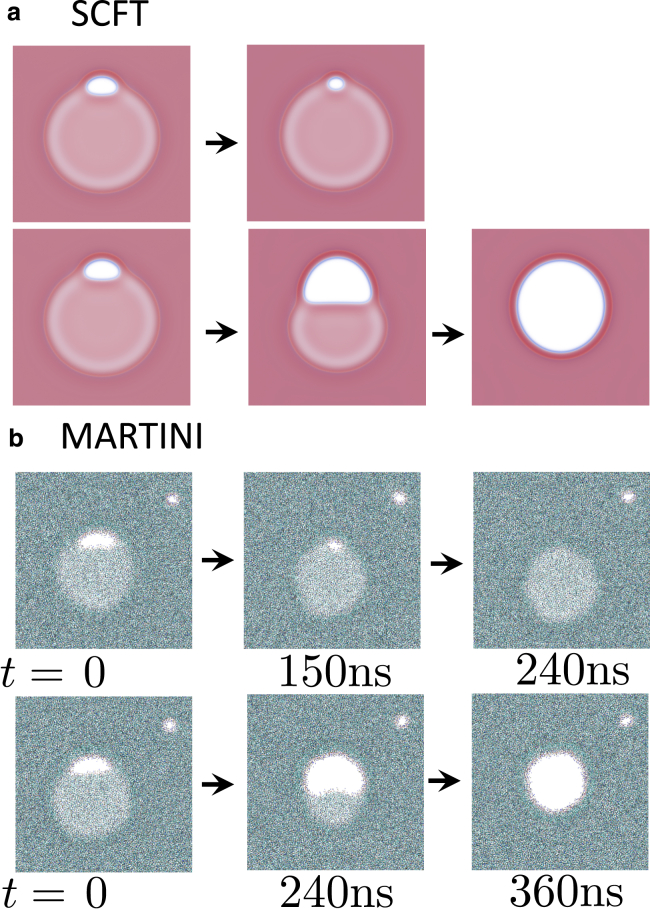
Progression of (*a*) SCFT at χN=30 and f=0.8 and (*b*) molecular dynamics simulations of DMPC membranes started with (*top*) subcritical and (*bottom*) supercritical pores, which therefore shrink or grow, respectively. The molecular dynamics simulations were both initialized by creating a pore of size $A_{p}=0.18A_{h0}\left(d\right)$ but small fluctuations lead to the pore growing or shrinking to become super- or subcritical. The auxiliary pores on the top-right corner allow for lipid flip-flop in the molecular dynamics simulation. To see this figure in color, go online.

Turning our attention to rim pores in circular HDs, once again, we utilize SCFT and molecular dynamics simulations to test the predictions of the phenomenological model. In both cases, we initialize HDs with rim pores that are either above or below the critical size and allow the system to relax. The results are shown in Fig. 8. In both cases, we find that large pores grow into a fusion pore, whereas small ones shrink, i.e., critical pores do exist. The critical size in SCFT, $A_{p}/A_{h0}\left(d\right)\approx 0.12$ is close to the predicted area fraction of approximately 0.14 for the given line tensions. Fluctuations, present in the molecular dynamics simulation, make it more difficult to bracket the critical size; however, our estimate of $A_{p}/A_{h0}\left(d\right)\approx 0.18$ is just below the predicted ≈0.2. In SCFT the pores shrink into a metastable pre-rim pore, whereas in the molecular dynamics simulations, rim pores eventually shrink away completely, as we saw for the linear interface. Alternatively, as shown in the movie in the supporting material, a rim pore may shrink into a metastable pre-pore, which then fluctuates in size until it happens to become supercritical and then grows into a fusion pore. This is possible due to the small critical size of rim pores.

**Figure 9:**
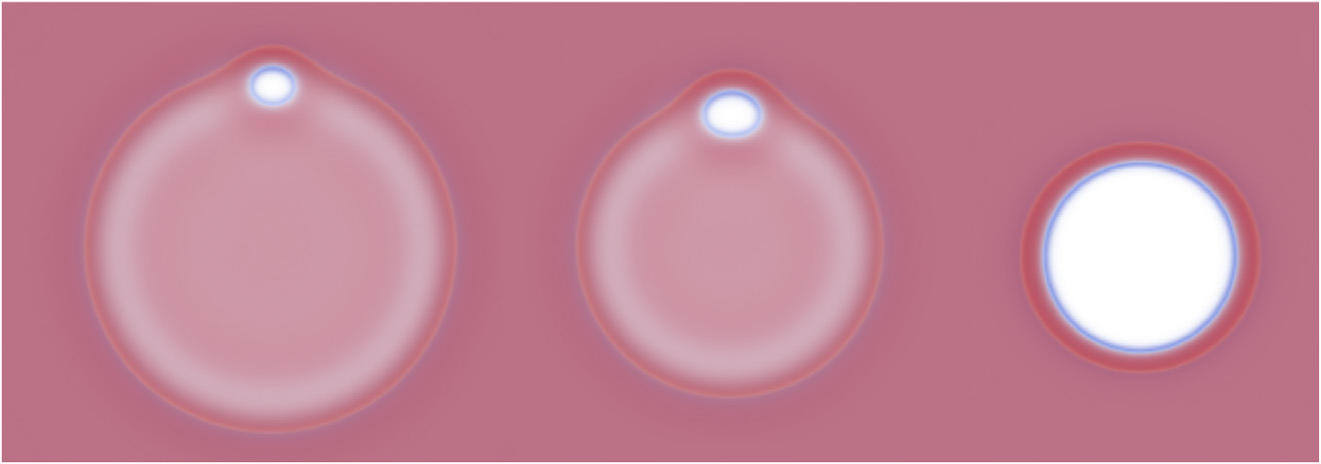
Pre-rim-pore behavior in circular HDs, obtained by SCFT. Large HDs may contain small, metastable pre-rim pores, similar in size to the zero-tension case. In smaller HDs (higher tension) the pre-rim pore grows. Even smaller HDs become unstable to transformation into a full fusion pore. To see this figure in color, go online.

The above analysis concerns the behavior of large pores in large HDs, and mostly ignores small metastable pre-rim pores, i.e., subcritical rim pores that cannot close completely because of the headgroup repulsion across the rim pore. This headgroup repulsion can be equivalently phrased as a dependence of the line tension on curvature. As we consider smaller HDs, like those we expect during SV fusion, we see that the pre-rim pore grows slightly: as the curvature increases, the fraction of the pre-rim pore circumference composed of the fusion-pore-like line segment increases. This line segment has a lower line tension and also a higher head density than the membrane-edge-like boundary, leading to a larger pre-rim pore. This is illustrated in Fig. 9. As we shrink the HD and grow the pre-rim pore, the pre-rim pore itself exceeds the critical rim pore size and grows, forming a fusion pore. Recall that the critical size is given as a fractional area of the HD. For our choice of parameters, the critical HD size is $R_{h0}\approx 3.75D\approx 15$ nm, below which it becomes unstable to pre-rim pore growth.

### Pores in fixed-size HDs

In marked contrast to pores in a single membrane in the NVT ensemble, rim pores are critical structures. The most negative eigenvalue of the Hessian chiefly corresponds to a change of the HD radius. Conceptually, the adjustment of the HD size enables sub- or supercritical rim pores to shrink or grow, respectively, without significantly altering the total membrane area or the membrane tension; the apposing membranes act like a membrane-area reservoir that is coupled to the HD. Thus, the instability of rim pores in the NVT ensemble is analogous to the instability of pores in a single membrane when the single-membrane tension is controlled (μVT- or NPT ensemble).

The next step is to attempt to stabilize rim pores by constraining the size of the HD. Fixing the HD radius, we remove the effective membrane-area reservoir and make the situation more analogous to a single-membrane pore in the NVT ensemble, i.e., at fixed number of lipids and membrane area, which results in a stable pore (73).

**Figure 10:**
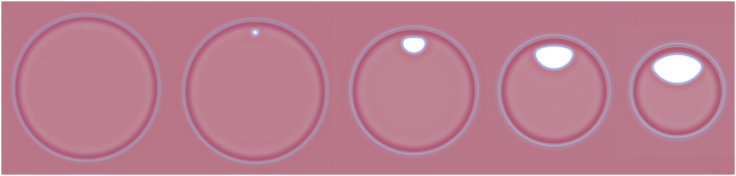
Images of HDs held at fixed radii using a ring, which wraps around them. Each calculation was done with the same number of lipids in the NVT ensemble. The leftmost image illustrates an equilibrium-sized HD, whereas the others are constricted by the ring. To see this figure in color, go online.

In SCFT, we can restraint the size of the HD, by inserting a ring around the HD, limiting its ability to grow. This also restricts the geometry of the rim pore as it cannot protrude out of the ring. Examples are shown in Fig. 10. Since the membrane area is (approximately) fixed, the size of the pore is dictated by the size of the ring.

Fixing the number of lipids, in experiment and in SCFT, does not fix the membrane area exactly, due to the area compressibility of the membrane. In the asymptotic limit of an infinite apposing-membrane reservoir, with fast lipid diffusion, any finite compressibility would destroy this effect. Real systems are, however, finite; lipid diffusion in a crowded membrane is slow, and the exchange of lipids between the HD and the apposing bilayers is additionally hindered by proteins that line the HD (77,78,79,80,81), mechanically restraining its radius. We thus expect results more like what we have shown in SCFT.

**Figure 11:**
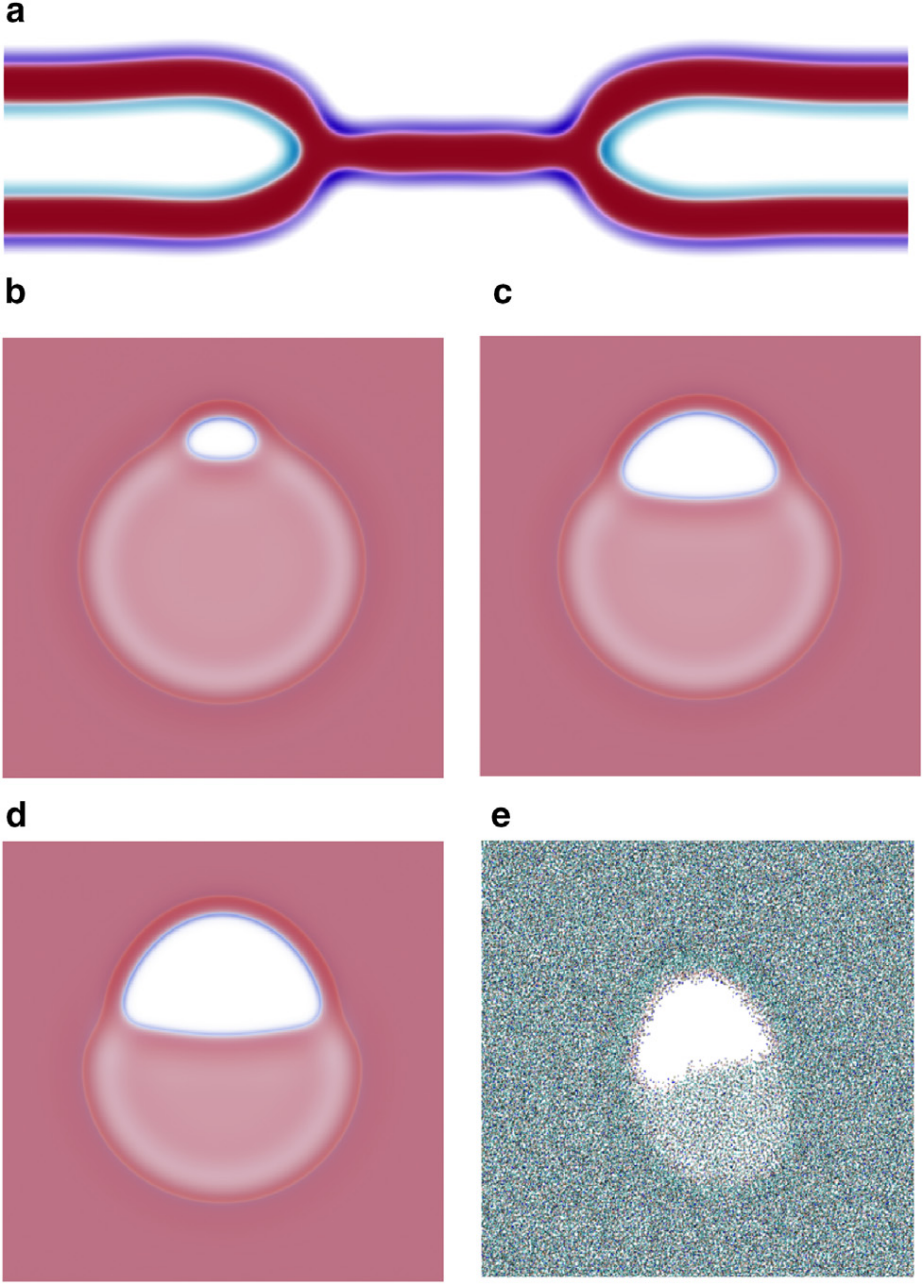
HDs with rim pores are shown for systems without flip-flops for (*a–d*) SCFT and (*e*) molecular dynamics simulations. Flip-flops are forbidden in SCFT through a repulsion between headgroups on the inner *cis* (*a*, *light blue*) and outer *trans* (*a*, *dark blue*) leaflets. An HD cross section is illustrated in (*a*) followed by (*b–d*) membranes with different numbers of lipids in the two, outer *trans* leaflets. Small and larger rim pores (*b* and *d*) are easily stabilized in SCFT, due to the lack of fluctuations. To see this figure in color, go online.

An alternate method for, effectively, constraining the HD size is to limit the flip-flop between the *trans* and *cis* leaflets of the apposing membranes, preventing them from exchanging area. In SCFT we can suppress lipid flip-flops between the disjunct leaflets by populating the *trans* and *cis* leaflets with two distinct types of lipids that are structurally identical except for a repulsion between their heads. In molecular dynamics simulation, lipid flip-flop is protracted if it is not purposely enhanced by an auxiliary pore, whose rim connects the leaflets. Snapshots from both approaches are shown in Fig. 11. As in the case of a straight three-bilayer junction, shown in Fig. 7, a rim pore is stabilized: the outer *trans* leaflets cannot use the inner *cis* ones as an effective lipid reservoir, thus the rim pore, which is topologically simply a pore in the outer leaflets, becomes stable. This is similar to the case of a pore in the canonical NVT ensemble. It remains a rim pore as $\lambda_{p}<\lambda_{e}+\lambda_{h}$, i.e., detaching the rim pore by moving it into the center of the HD does not reduce the free energy.

### Rim pore formation and growth

Controlling the flow of neurotransmitters through a rim pore requires control over the size of the pore: it must form, grow to a sufficient area, allow the passage of material, and then, in the case of the K&R mechanism, shrink away. We therefore turn our attention to the free-energy barriers of the formation of rim pores and their growth into fusion pores.

**Figure 12:**
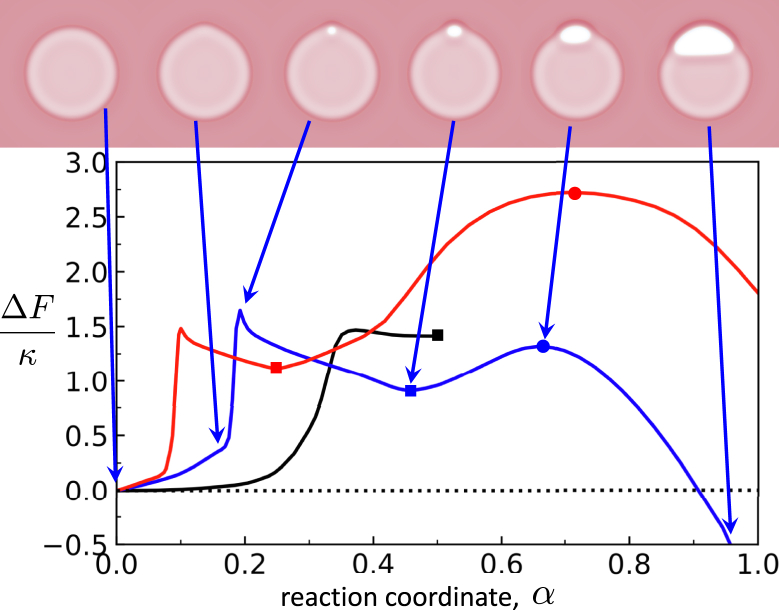
Free energy along the minimum free-energy pathway for the formation of a rim pore and its growth into a fusion pore. The metastable pre-rim pore and the critical rim pore are indicated by a square and a circle, respectively. Data are shown for a rim pore forming on a linear three-bilayer junction (*black*) at zero tension, corresponding to the limit as the HD radius diverges (see Fig. 7), in a small circular HD (*blue*) with $R_{h0}\approx 20\;\text{nm}$, and a larger circular HD (*red*), with $R_{h0}\approx 30\;\text{nm}$. $R_{h0}$ denotes the radius of the HD *without* rim pore. Key steps, i.e., metastable states (pre-rim pores) and saddlepoints (critical rim pores), are shown for the small circular HD with arrows indicating the point along the path corresponding to each image. Free energies are given in units of the bilayer bending modulus, κ. Pore growth is not shown for the linear, three-bilayer junction, as the pore never becomes stable. To see this figure in color, go online.

SCFT, combined with the string method (42), allows us to examine the optimal pathway for rim pore formation and growth. The free energy and key steps along the minimum free-energy path from a metastable HD, α=0, toward a fusion pore are presented in Fig. 12: first, a bulge forms in the HD rim. The membrane within this bulge then thins and ruptures, forming a pore. The pore then relaxes into a metastable pre-rim pore. As the rim pore grows, the free energy increases until it reaches its critical size. This is the state predicted by the phenomenological model. After this critical size, as the rim pore grows further, the free energy decreases. Note that the free-energy difference, ΔF, of the fusion pore with respect to the HD is negative because $\lambda_{p}<\lambda_{h}$, and, also, the radius of the HD is larger than the radius of the fusion pore in the NVT ensemble. Qualitatively, this heterogeneous nucleation of a membrane pore at the rim of the HD in the canonical NVT ensemble is similar to the nucleation of the single-membrane pore at constant membrane tension or chemical potential (27,62,76), indicating that the adjustment of the HD radius effectively acts like a reservoir.

The barrier to nucleate an initial pore at the bulged rim of the HD is on the order to $1.5\kappa \approx 30k_{B}T$; somewhat large but not insurmountable. Given the large area over which these initial pores may form, we expect them to readily come into existence around the rim of the HD. The nucleation rate is not entirely described by the free energy barrier, as it also depends on a kinetic prefactor (the rate of “attempting” to nucleate). Determining the factor is beyond the scope of this work, but can be accomplished through techniques such as forward-flux sampling (82). After nucleating an initial pore at the rim, its size expands to a metastable pre-rim pore. The barrier to closing this pre-rim pore, however, is quite small—on the scale of $k_{B}T$. This is one reason why we only observe short-lived metastable pre-rim pores in the molecular dynamics simulations.

The barrier to grow from the metastable pre-rim pore to a fusion pore increases with HD size, as expected from the phenomenological model (note the $R_{h0}$ scaling of the free energy) and vanishes when the pre-rim pore is the size of the critical rim pore, as described previously. Growth of the pre-rim pore, as we established previously, requires exchange of lipids, either between the leaflets or with membrane regions far from the HD. This should, effectively, decrease the kinetic prefactor of the Arrhenius rate of barrier crossing, depending on the lipid diffusion rate.

The picture that we obtain from this description is that of flickering pre-rim pores that readily open and close. Since the pre-rim pore size is limited, each such pre-rim pore may allow the passage of small amounts of neurotransmitter, consistent with previous descriptions of the K&R mechanism (12,17,18,19). There are alternate mechanisms available to cellular systems to keep the pores open longer. These mechanisms and their relationship to the equilibrium description provided earlier will be discussed in the following.

## Discussion

The formation and poration of HDs provides a mechanism by which material may be transferred across two membrane bilayers, for example, during the release of neurotransmitter from a SV to outside of the presynapse (1,10,11). To achieve this, biological systems must carefully control the tendency for any pores that form to shrink away, obviating the transfer of material, and the tendency for pores to grow unbounded, producing a large fusion pore and complicating the reverse process, endocytosis. If the mechanism includes full fusion followed by clathrin-mediated endocytosis, this does not necessarily present a problem; however, there are cases where it is advantageous to close the pore and recover the vesicle, such as the K&R mechanism for neurotransmitter release (1,10,11).

Transferring material in this way requires pores that are both large enough and long-lived enough for neurotransmitters to pass through, while still being able to shrink away after the process. Our work identifies a window of lipid membrane properties that allow for the formation of rim pores and chemical and mechanical constraints to render rim pores metastable membrane structures. Although we focus on rim pores, we have identified a region in line tension space, $\lambda_{e}<\lambda_{p}+\lambda_{h}$, where the pores detached from the rim. Based on our simulations and previous observations of vesicle fusion (31,32,34), we expect rim pores to be more typical. Although technological limitations make direct observation of rim pores in SVs challenging, they have been observed in larger hemifused vesicles (31,32,34), and the flickering nature of the rim pore that we predict is consistent with previous observations of SVs (12,17,18,19).

Figs. 4 and 5 predict constraints on physical parameters where critical rim pores in the NVT ensemble are possible, in terms of the line tensions, $\lambda_{h}$, $\lambda_{e}$, and $\lambda_{p}$, of the relevant line segments of the rim pore and the distance between the two apposing membranes, 2d. It is already known that the control of line tensions can be used to manipulate membrane geometry and topology (83,84,85,86), and that this can be achieved through the action of proteins (85), changing lipid composition (87), or by the preferential segregation of other molecules, such as, e.g., cholesterol (86,88). The ability of cellular systems to control line tensions on the fly, moving between different regions in Figs. 4 and 5, would allow them to grow or shrink pores. In addition to opening and closing a rim pore, manipulating $\lambda_{h}$ also provides a mechanism to grow or shrink the HD before and after the transfer occurs.

In contrast to pores in single membranes in the NVT ensemble, rim pores are critical. To convert them into metastable structures, one needs to constrain lipid exchange, i.e., N= const, and *additionally* constrain variations of the HD radius.

Growing or shrinking pores entails the transport of lipids. Viewing the HD as two outer *trans* leaflets (*dark blue* in Fig. 11) sandwiching an inner *cis* leaflet (*light blue*), the pore is a topological structure in the outer *trans* leaflets. Lipids may either diffuse along the outer *trans* leaflets, using the bulk as an effective reservoir, or flip-flop across the membrane, using the inner *cis* leaflet as an effective reservoir and changing the size of the HD. Using the outer *trans* leaflets as an effective reservoir would require either protracted diffusion over long distances or hydrodynamic flow of the entire leaflet and may be additionally hindered by the presence of membrane proteins, acting like a “fence” (77,78,79,80,81).

The free-energy barrier to flip-flop across the membrane, however, is normally large, leading to a very slow process which requires catalysis by flipase or scramblase proteins, that are thought to be involved in HD dynamics. Without flipases, flip-flops occur at a rate of ≈10/ s (89), which results in about 1 flip-flop in our simulation ($10^{4}$ lipids) per 10μs. ATP-dependent flipases increase this rate by an order of magnitude, and Ca^2+^-dependent scramblases increase the rate by 2 to 3 orders of magnitude (90) and are thought to be involved in neurotransmission (91).

Sterols are also present in significant numbers (92), can flip-flop more readily (93), and may provide another avenue for leaflets to exchange area. However, even at these increased rates ($10^{3}-10^{4}$ per s) this can only account for a ≈0.1% change in system area per *μ*s. Fortunately, flipases are known to be present in a wide variety of biological contexts (94,95,96,97,98), and may thus provide a method to turn on and off flip-flops, and thus another strategy to control rim pores. Without flip-flops and with limited lipid exchange with the far-away bulk, pores may be stabilized long enough for neurotransmitter exchange, before flippase or scramblase action is re-enabled, allowing the pore to shrink away.

In addition to its effects on the pore, control of flip-flop rates may also be used to control the HD itself: constraining the number of lipids in each leaflet, through restricted flip-flops, mechanically constrains the radius of the HD by preventing the inner *cis* leaflet from changing in area and allowing for a difference in tension, and lipid chemical potential, between leaflets. This is another mechanism that could be used to hold the HD in place, while the SNARE proteins disengage. When flip-flops are re-enabled, the difference in chemical potential could cause the lipids to flow from the outer *trans* membrane into the inner *cis* membrane, closing the HD.

The discussion above provides a number of ways that cells can control the geometry of various structures that we combine into a potential mechanism for the K&R mechanism: 1) as initial step, SNARE proteins bring the vesicle to the cell membrane and initiate fusion, forming an HD. 2) Flickering pores, on the scale of approximately 5 nm, readily form at the rim of the HD, allowing the passage of a few neurotransmitter molecules. 3) The pore is partially opened, perhaps through the action of proteins or other molecules, which change line tensions. Our simulations suggest that this occurs on a timescale of tens of ns. Neurotransmitters must diffuse the length of the vesicle to exit, which occurs in approximately 100 ns (99). 4) SNAREs then release, and their disconnected transmembrane portions cannot exert forces on the membrane. Flipases are re-enabled, allowing the HD to close, which occurs in hundreds of ns to *μ*s. 5) The HD shrinks away and disconnects, perhaps aided by the action of dynamin, to perform the final scission.

The above mechanism requires a careful choreography of cellular machinery to dictate membrane behavior. Various proteins may be implicated in controlling the HD size, including SNAREs, which pull the membrane together and manipulate the geometry of the HD boundary (100,101,102,103,104,105); clathrin proteins (106,107,108,109,110) and actin (111), which act as a membrane scaffold; and dynamin, which forms helical structures around tubes and catalyses their fission (110,112). All of these classes of proteins have been implicated in the K&R mechanism, and it appears possible that they could be used in pore opening and closing. In addition to the K&R mechanism, the control mechanisms that we have described are applicable to a wide variety of problems involving HDs and pores.

We have treated membranes as consisting purely of lipids, ignoring the proteins and sterols, which are known to be present. Uniformly distributed molecules, such as sterols and lipids of different architectures, may alter bulk membrane properties, such as line tensions, and thus simply move the system from one point to another in the landscape of behavior that we have mapped out. There may be, however, heterogeneous molecular distributions. These include asymmetric distributions of lipid types between leaflets and within the same leaflet (lipid rafts), as well as heterogeneities in the distribution of proteins and sterols. This can affect HD and pore behavior if, for example, certain lipid species segregate to the HD rim, either through the adsorption of lipid rafts or through absorption of individual lipids of different types, bringing about changes in line tensions. While interesting, this effect is outside the scope of this work.

Our description of an HD as being between two parallel apposing bilayers does not consider the influence of vesicle curvature on the geometry of the membrane-vesicle connection: see, for example, the difference between the depictions shown in Figs. 1 and 2. The main effect that we anticipate is on the line tensions, as there is an increase in the “height” of the interface (effectively an increase in *d*). The effect on the line tensions is to decrease $\lambda_{p}$ and (less so) $\lambda_{h}$. In terms of our predictions for rim pore stability described in Fig. 4, $\lambda_{e}/\lambda_{h}$ increases due to the decrease in $\lambda_{h}$ and $\lambda_{p}/\lambda_{h}$ decreases, as $\lambda_{p}$ decreases faster than $\lambda_{h}$. A point in Fig. 4 would thus be shifted “up” and “left,” i.e., the critical pore is likely to get smaller and more likely to protrude outward. Investigations involving the entire vesicle and effects thereof are left for future work.

## Conclusion

This work has combined molecular dynamics simulations, field-based lipid calculations, and phenomenological membrane modeling to study the mechanics of rim pores in HDs. We find that pores readily form in HDs, and we study their stability in detail, presenting a number of mechanisms, by which large pores may be formed and stabilized to allow the transfer of material across apposing membranes.

The primary factors controlling the stability and growth of pores are the line tensions of the distinct line segments of the rim pore and the transport of lipids between the HD and the *trans* and *cis* leaflets of the apposing membranes. If lipids are allowed to cross the membrane, flip-flop, and the HD is allowed to change in size, pores are always unstable. The line tensions control the nature of this stability, i.e., whether rim pores shrink or grow. Rim pores exhibit a critical size, below which they shrink and above which they grow. If cellular systems can control these line tension, they can thereby cause rim pores to switch between tendencies to grow or shrink.

The ability for lipids to flip-flop creates an effective reservoir. Turning on or off flip-flops thus makes the pore behave more like a pore in the grand canonical or canonical ensemble, respectively, i.e., grow/shrink unbounded or stabilize to a given size. Turning on or off flip-flops thus provides a switch that may be toggled to make rim pores unstable or stable. The possibility of restricting the HD size provides another level of control, as this can restrict the effective reservoir or change its chemical potential, controlling whether rim pores grow or shrink.

This work suggests that cellular systems have multiple ways by which they can control the growth, shrinking, and stability of pores in HDs, and we propose a step-by-step mechanism by which neurons may use these mechanisms to facilitate the K&R mechanism for synaptic neurotransmitter release. Beyond this particular application, this plethora of possibilities opens the door to many actionable avenues of research into how cellular systems move material across membranes.

## Author contributions

R.S. performed the SCFT calculations, some of the phenomenological calculations, and wrote the initial draft. Y.S. performed some of the phenomenological calculations and some of the MARTINI simulations. A.S. performed some of the MARTINI simulations. M.M. designed the research. All authors discussed the results and approved the final version of the manuscript.

## Figures and Tables

**Figure 13:**
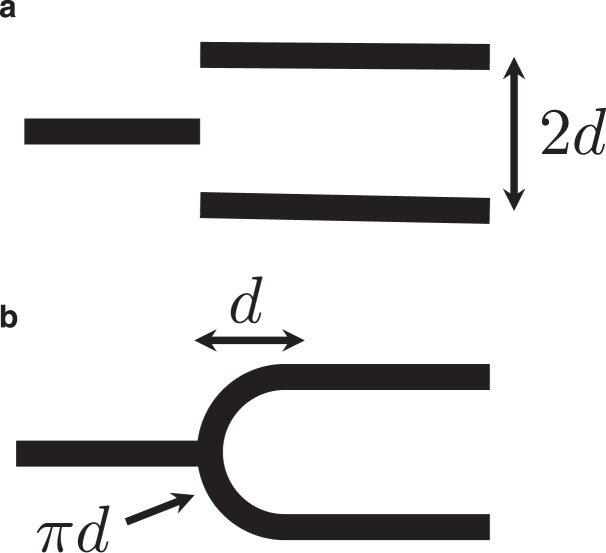
Sketch of the area deviation due to the membrane separation, 2d, for the threefold junction. The simple description ignores the membrane area used to connect the different regions, i.e., it treats the threefold junction as depicted in (*a*). A more realistic scenario is (*b*), where a semicircular membrane region of length πd replaces a membrane distance of 2d (1 *d* for each membrane in the double layer).

**Figure 14:**
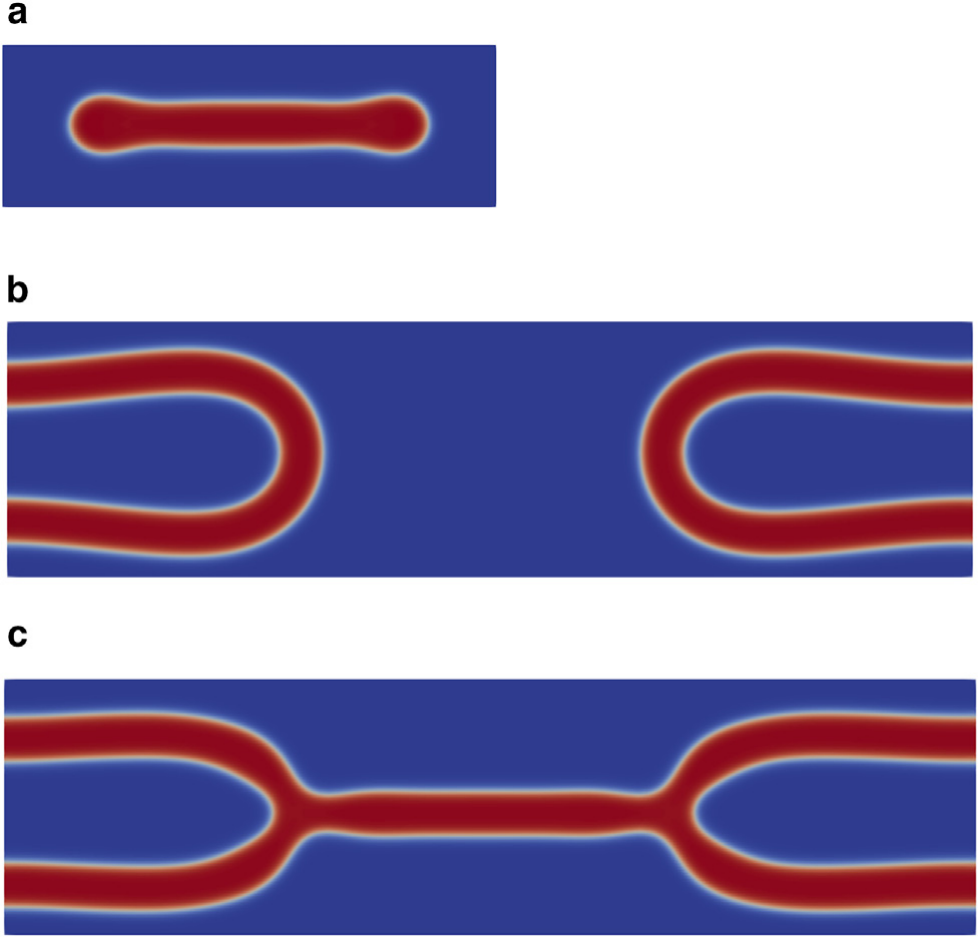
Example systems used in SCFT calculations to obtain the line tensions for (*a*) edge, *e*, (*b*) rim pore, *p*, and (*c*) HD, *h*, interfaces. Double-layer separation, *d*, is controlled using periodic boundaries and controlling the system size. Hydration repulsion prevents the double-layer region from expanding into a circular cross section. *d* is chosen to match the double-layer region, as described further in Fig. 3. To see this figure in color, go online.

**Figure 15:**
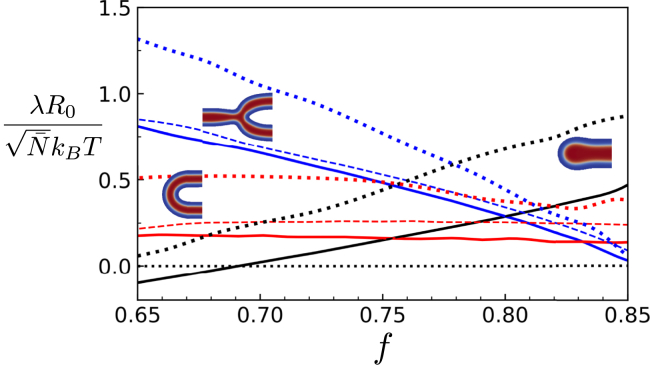
Absolute line tensions corresponding to Fig. 3, in units of $\sqrt{\bar{N}}k_{B}T$. Data are shown for $\lambda_{e}$ (*black*), $\lambda_{p}$ (*red*), and $\lambda_{h}$ (*blue*). Calculations are done for χN=30 and 2d≈2.75D≈11nm (*solid*), χN=30 and 2d≈1.75F≈7nm (*dashed*), and χN=45 and 2d≈2.75D≈11nm (*dotted*). The parameters of primary interest, f=0.8 and χN=30, have line tensions of $\lambda_{e}=0.288\sqrt{\bar{N}}k_{B}T/R_{0}$, $\lambda_{h}=0.290\sqrt{\bar{N}}k_{B}T/R_{0}$, and $\lambda_{p}=0.160\sqrt{\bar{N}}k_{B}T/R_{0}$. The absolute line tensions found in MARTINI simulations are POPC: $\lambda_{e}=\left(14.5\pm 0.5\right)k_{B}T/\text{nm}$, $\lambda_{h}=\left(11.5\pm 1.0\right)k_{B}T/\text{nm}$, $\lambda_{p}=\left(18.3\pm 1.5\right)k_{B}T/\text{nm}$, and 2d=4.7±0.1 nm; DMPC: $\lambda_{e}=\left(9.4\pm 0.5\right)k_{B}T/\text{nm}$, $\lambda_{h}=\left(16.5\pm 1.0\right)k_{B}T/\text{nm}$, $\lambda_{p}=\left(17.5\pm 1.3\right)k_{B}T/\text{nm}$, and 2d=4.3±0.1 nm. For comparison between SCFT and particle simulations or experiments, $\sqrt{\bar{N}}k_{B}T\approx 4.78\kappa \approx 96k_{B}T$ and $R_{0}\approx 0.83D\approx 3.3$ nm. To see this figure in color, go online.

**Figure 16:**
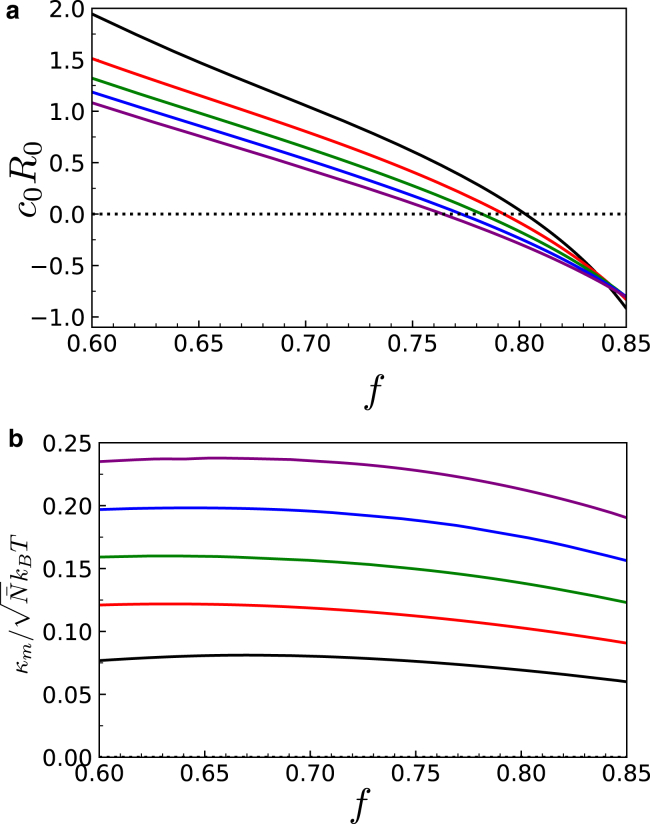
(*a*) Monolayer spontaneous curvature, $c_{0}$, and (*b*) monolayer bending energy, $\kappa_{m}$, are shown as functions of lipid tail volume fraction, *f*, for χN=25 (*black*), χN=30 (*red*), χN=35 (*green*), χN=40 (*blue*), and χN=45 (*purple*). To see this figure in color, go online.

**Figure 17:**
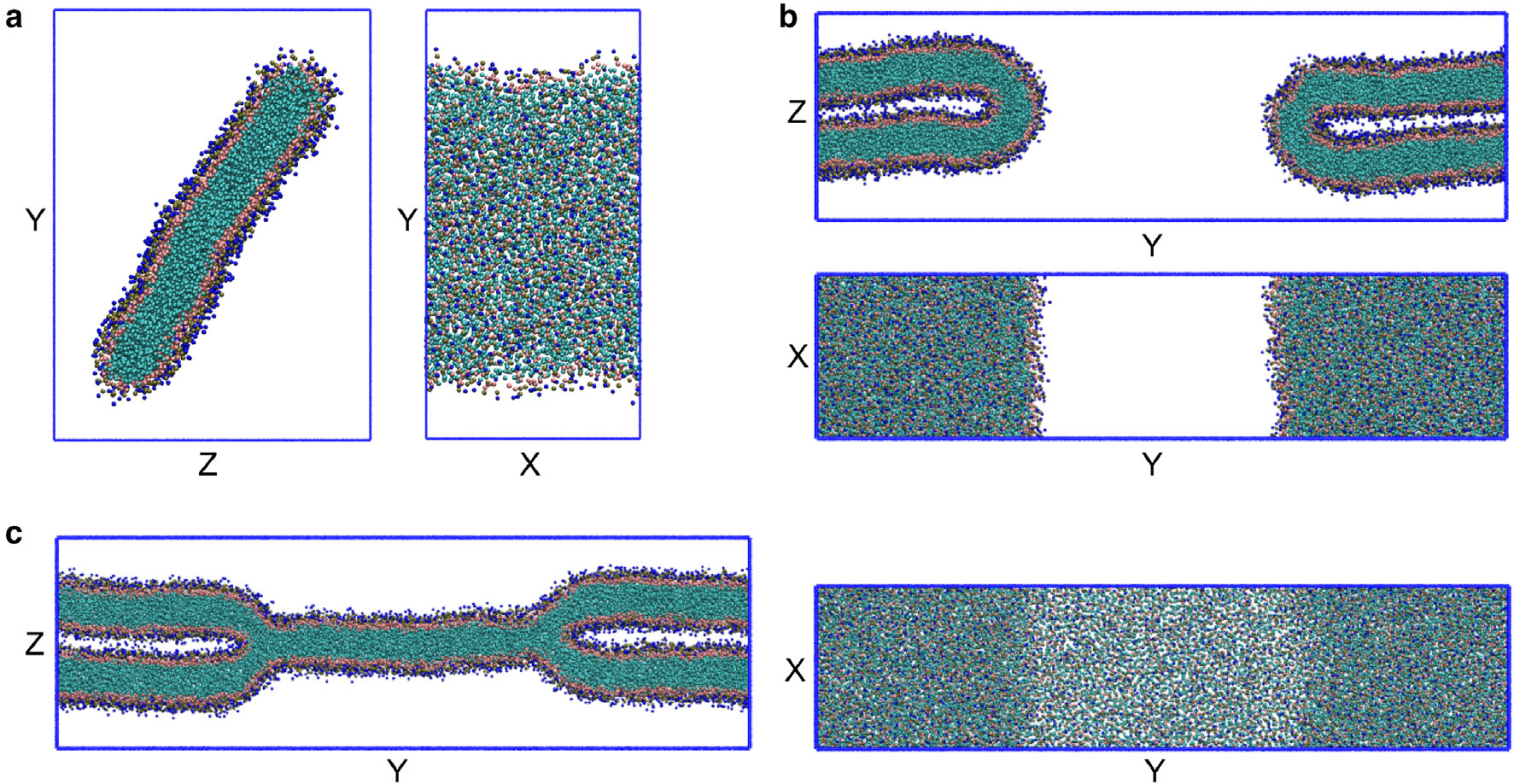
Example systems used in MARTINI simulations to calculate the line tensions for (*a*) edge, *e*, (*b*) rim pore, *p*, and (*c*) HD, *h*, interfaces. The separation of the double-layer regions, *d*, is controlled by the amount of water contained between the layers, as diffusion of water across the membrane is slow on the simulation timescale. *d* is chosen to match that chosen by the circular HD systems. To see this figure in color, go online.

**Table 1:** MARTINI systems for line tension calculation and verification of DMPC simulations

System	Size (nm^3^)	No. of lipids	No. of water beads
Membrane edge	10.98×22.09×16.02	655	26,771
HD edge	11.58×48.73×15.04	3040	44,274
Fusion pore edge	11.58×48.71×14.76	2477	47,898
HD	43.90×46.10×17.86	12,516	192,390
Fig. 8*b*	43.52×45.70×18.12	12,388	192,390
Fig. 11*e*	43.55×45.73×18.05	12,291	192,390

## Appendix A: Phenomenological model

Equilibrium pores and HDs balance the contractile force from their line tensions and the expansive force from the membrane tension. This is analogous to the surface tension and pressure in bubbles, and gives rise to a similar Laplace condition. For a membrane under tension Σ, an equilibrium pore with line tension $\lambda_{e}$ stabilizes at a radius $R_{e}=\lambda_{e}/\Sigma$ (73). For an HD (*h*) or fusion pore (*p*), the membrane tension is replaced by the difference in tension inside versus outside leading to $R_{h}=\lambda_{h}/\Sigma$ and $R_{p}=\lambda_{p}/2\Sigma$. This balance of forces dictates the constant radii of curvature for the intersecting circular arcs that describe the HD and pore system, as shown in Fig. 2. At the intersection points, the Neumann force balance ensures a net force of zero, reflecting the system’s mechanical stability.

To quantitatively understand the stability of rim pores in HDs, we analytically derive the free energy of a rim pore in an HD, with geometry summarized in Fig. 2. A system, with area $A_{\text{sys}}$, contains a membrane of area $A_{m}$ and has a membrane tension Σ. The membrane forms a double layer outside of the HD and a single layer inside the HD of area $A_{h}$, and the pore has an area $A_{p}$. These areas must satisfy $A_{m}=2A_{\text{sys}}-2A_{p}-A_{h}$.

The free energy associated with inserting the pore is

(Equation 1) $$\Delta F=2\pi \lambda_{h}R_{h}+2\lambda_{e}R_{e}\;\sin^{-1}\left(a/R_{e}\right)+2\lambda_{p}R_{p}\;\sin^{-1}\left(a/R_{p}\right)\;-2\lambda_{h}R_{h}\;\sin^{-1}\left(a/R_{h}\right)\;+2\lambda_{e}\Lambda_{e}R_{e}\left(\pi -2\;\sin^{-1}\left(a/R_{e}\right)\right)\;+2\lambda_{p}\Lambda_{p}R_{p}\left(\pi -2\;\sin^{-1}\left(a/R_{p}\right)\right)\;+\Sigma \left(2A_{\text{sys}}-2A_{p}-A_{h}-A_{m}\right)$$

where *a* is the half-width of a pore, and we treat Σ as a Lagrange multiplier, enforcing the area constraint above. When no pore is present, this reduces to the expected form, $\Delta F=2\pi \lambda_{h}R_{h}^{2}+\Sigma \left(2A_{sys}-\pi R_{h}^{2}\right)$. An equilibrium HD ($\partial_{R_{h}}\Delta F=0$) thus has radius $R_{h0}=\lambda_{h}/\Sigma$ as expected. The $\Lambda_{\gamma }$ terms (γ=e, *p* or *h*) allow for the possibility that the pore may “protrude” (see illustrations in Fig. 4) and $\Lambda_{\gamma }=1$ if the γ interface protrudes and 0 otherwise. The pore is defined as “protruding” outward when the *p* arc represents a major arc of its defining circle and “recessed” when the arc subtends a minor segment. Protruding and recessed inward are defined similarly for the *e* arc. We assume that the line tensions are fixed, having no dependence on the curvature of the interfaces. A more complete description could include this curvature dependence, but is beyond the scope of our calculations.

We can calculate the areas, $A_{p}$ and $A_{h}$ from the geometries shown in Fig. 2, yielding the area constraint

(Equation 2) $$A_{m}=2A_{\text{sys}}-\pi R_{h}^{2}\;+a\left(2\sqrt{R_{p}^{2}-a^{2}}+\sqrt{R_{e}^{2}-a^{2}}-\sqrt{R_{h}^{2}-a^{2}}\right)\;-\Lambda_{e}\left(\pi R_{e}^{2}-2R_{e}^{2}\;\sin^{-1}\left(a/R_{e}\right)+2a\sqrt{R_{e}^{2}-a^{2}}\right)\;-2\Lambda_{p}\left(\pi R_{p}^{2}-2R_{p}^{2}\;\sin^{-1}\left(a/R_{p}\right)+2\tilde{a}\sqrt{R_{p}^{2}-a^{2}}\right)\;+R_{h}^{2}\;\sin^{-1}\left(a/R_{h}\right)-2R_{p}^{2}\;\sin^{-1}\left(a/R_{p}\right)\;-R_{e}^{2}\;\sin^{-1}\left(a/R_{e}\right)\text{.}$$

The geometry in the above formulation is invariant to the scale of the membrane. That is, if we scale all lengths by the same quantity, and scale Σ appropriately, the physics remains the same. We therefore define a length $R_{h0}=\sqrt{A_{h0}/\pi }$, where $A_{h0}=2A_{\text{sys}}-A_{m}=\pi R_{h0}^{2}$ is the HD area with no pore present, i.e., $R_{h0}$ is the radius of an unperturbed (no pore) HD. This allows us to define ${\tilde{R}}_{\gamma }=R_{\gamma }/R_{h0}$. Similarly, we can scale out a constant from the line tensions. For this we choose to define ${\tilde{\lambda }}_{\gamma }=\lambda /\lambda_{h}$. The resulting scaled free energy is

(Equation 3) $$\begin{array}{ll} \frac{\Delta F}{\lambda_{h}R_{h0}} & =2\pi {\tilde{R}}_{h}+2{\tilde{\lambda }}_{e}{\tilde{R}}_{e}\;\sin^{-1}\left(\tilde{a}/{\tilde{R}}_{e}\right)+2{\tilde{\lambda }}_{p}{\tilde{R}}_{p}\;\sin^{-1}\left(\tilde{a}/{\tilde{R}}_{p}\right)-2{\tilde{R}}_{h}\;\sin^{-1}\left(\tilde{a}/{\tilde{R}}_{h}\right)\\ & +2\lambda_{e}\Lambda_{e}{\tilde{R}}_{e}\left(\pi -2\;\sin^{-1}\left(\tilde{a}/{\tilde{R}}_{e}\right)\right)+2\lambda_{p}\Lambda_{p}{\tilde{R}}_{p}\left(\pi -2\;\sin^{-1}\left(\tilde{a}/{\tilde{R}}_{p}\right)\right)\\ & +R_{h0}\frac{\Sigma }{\lambda_{h}}[\pi -\pi {\tilde{R}}_{h}^{2}+\tilde{a}\left(2\sqrt{{\tilde{R}}_{p}^{2}-{\tilde{a}}^{2}}+\sqrt{{\tilde{R}}_{e}^{2}-{\tilde{a}}^{2}}-\sqrt{{\tilde{R}}_{h}^{2}-{\tilde{a}}^{2}}\right)\\ & +{\tilde{R}}_{h}^{2}\;\sin^{-1}\left(\tilde{a}/{\tilde{R}}_{h}\right)-2{\tilde{R}}_{p}^{2}\;\sin^{-1}\left(\tilde{a}/{\tilde{R}}_{p}\right)-{\tilde{R}}_{e}^{2}\;\sin^{-1}\left(\tilde{a}/{\tilde{R}}_{e}\right)\\ & -\Lambda_{e}\left(\pi {\tilde{R}}_{e}^{2}-2{\tilde{R}}_{e}^{2}\;\sin^{-1}\left(\tilde{a}/{\tilde{R}}_{e}\right)+2\tilde{a}\sqrt{{\tilde{R}}_{e}^{2}-{\tilde{a}}^{2}}\right)\\ & -2\Lambda_{p}\left(\pi {\tilde{R}}_{p}^{2}-2{\tilde{R}}_{p}^{2}\;\sin^{-1}\left(\tilde{a}/{\tilde{R}}_{p}\right)+2\tilde{a}\sqrt{{\tilde{R}}_{p}^{2}-{\tilde{a}}^{2}}\right)] \end{array}$$

To optimize the free energy, we split Eq. 3 into

(Equation 4) $$\begin{array}{cc} \frac{\Delta F}{\lambda_{h}R_{h0}} & =2\pi {\tilde{R}}_{h}+2{\tilde{\lambda }}_{e}{\tilde{R}}_{e}\;\sin^{-1}\left(\tilde{a}/{\tilde{R}}_{e}\right)+2{\tilde{\lambda }}_{p}{\tilde{R}}_{p}\;\sin^{-1}\left(\tilde{a}/{\tilde{R}}_{p}\right)-2{\tilde{R}}_{h}\;\sin^{-1}\left(\tilde{a}/{\tilde{R}}_{h}\right)\\ & +2\lambda_{e}\Lambda_{e}{\tilde{R}}_{e}\left(\pi -2\;\sin^{-1}\left(\tilde{a}/{\tilde{R}}_{e}\right)\right)+2\lambda_{p}\Lambda_{p}{\tilde{R}}_{p}\left(\pi -2\;\sin^{-1}\left(\tilde{a}/{\tilde{R}}_{p}\right)\right) \end{array}$$

(Equation 5) $$\begin{array}{cc} C_{A} & =\pi -\pi {\tilde{R}}_{h}^{2}\\ & +\tilde{a}\left(2\sqrt{{\tilde{R}}_{p}^{2}-{\tilde{a}}^{2}}+\sqrt{{\tilde{R}}_{e}^{2}-{\tilde{a}}^{2}}-\sqrt{{\tilde{R}}_{h}^{2}-{\tilde{a}}^{2}}\right)\\ & +{\tilde{R}}_{h}^{2}\;\sin^{-1}\left(\tilde{a}/{\tilde{R}}_{h}\right)-2{\tilde{R}}_{p}^{2}\;\sin^{-1}\left(\tilde{a}/{\tilde{R}}_{p}\right)-{\tilde{R}}_{e}^{2}\;\sin^{-1}\left(\tilde{a}/{\tilde{R}}_{e}\right)\\ & -\Lambda_{e}\left(\pi {\tilde{R}}_{e}^{2}-2{\tilde{R}}_{e}^{2}\;\sin^{-1}\left(\tilde{a}/{\tilde{R}}_{e}\right)+2\tilde{a}\sqrt{{\tilde{R}}_{e}^{2}-{\tilde{a}}^{2}}\right)\\ & -2\Lambda_{p}\left(\pi {\tilde{R}}_{p}^{2}-2{\tilde{R}}_{p}^{2}\;\sin^{-1}\left(\tilde{a}/{\tilde{R}}_{p}\right)+2\tilde{a}\sqrt{{\tilde{R}}_{p}^{2}-{\tilde{a}}^{2}}\right) \end{array}$$

where the second equation is the deviation of the area from the desired area. Given line tensions, $\lambda_{e}$, $\lambda_{p}$, and $\lambda_{h}$, and an equilibrium HD radius, $R_{h0}$, this free energy is a function of four variables, ${\tilde{R}}_{e}$, ${\tilde{R}}_{p}$, ${\tilde{R}}_{h}$, and $\tilde{a}$, which define the geometry of the pore. Fixing $C_{A}=0$, Eq. 5 reduces this dependence to three variables. We choose to solve for $\tilde{a}$, which is done numerically.

Optimizing ΔF under the constraint $C_{A}=0$ entails solving the system of equations

(Equation 6) $$\frac{d\Delta F}{d{\tilde{R}}_{\gamma }}=\frac{\partial \Delta F}{\partial {\tilde{R}}_{\gamma }}-\frac{\partial \Delta F}{\partial \tilde{a}}\frac{\partial C_{A}}{\partial {\tilde{R}}_{\gamma }}{\left(\frac{\partial C_{A}}{\partial \tilde{a}}\right)}^{-1}=0\text{.}$$

Each term in Eq. 6 can be calculated analytically, but, to solve it, we still need to find $\tilde{a}$, i.e., solve $C_{A}=0$, numerically. We thus solve Eq. 6 numerically.

The solutions correspond to configurations where there is a net zero force everywhere, particularly on the contact points between the pore and HD, i.e., the junctions of different color lines in Fig. 2. Looking at it this way, it becomes clear why the system is scale invariant: multiplying all lengths or all line tensions by a constant does not change the angles or the resulting force balance conditions required for a stable configuration. Scaling the membrane tension by $R_{h0}/\lambda_{h}$ accounts for the change in the radii of curvature of the interfaces.

Once we have found the optima, we want to know if they are stable or unstable optima, i.e., metastable or critical pores. To do this, we calculate the Hessian matrix, $H_{i,j}=\partial^{2}\Delta F/\partial R_{i}\partial R_{j}$. An eigenvalue decomposition gives us the principle curvatures of the free energy. If at least one eigenvalue is negative, the configuration is unstable and the corresponding eigenvector tells us the “direction” in $R_{h}-R_{e}-R_{p}$ space that the instability corresponds to. If all eigenvalues are positive, then the configuration is metastable. We can look for conditional stability, for example, fixing one of the parameters, by simply excluding it from the Hessian matrix. More sophisticated methods are available (113) but are not necessary for our problem.

This description works well for large HDs, where the length scales of the HD geometry are large compared with the separation of the membranes comprising the double layer. For smaller HDs, a discrepancy arises because some of the membrane area goes toward forming the connection between the double layer and the single layer (in the *h*-type interface) and in connecting the two layers (in the *p*-type interface). This is illustrated for the *h*-type in Fig. 13. The result is an extra membrane area of (π−2)d times the length of the *h*- and *p*-type interfaces.

This modifies our calculation by adding the following terms to $C_{A}$

(Equation 7) $$2\left(\pi -2\right)\tilde{d}\left({\tilde{R}}_{p}\;\sin^{-1}\left(\tilde{a}/{\tilde{R}}_{p}\right)+{\tilde{R}}_{h}\left(\pi -\sin^{-1}\left(\tilde{a}/{\tilde{R}}_{h}\right)\right)\right)$$

where $\tilde{d}=d/R_{h0}$. To take this correction into account, we follow the same procedure outlined above, with different values of $\tilde{d}$. Note that our approximation of the connection as a semicircle is not exact, but other geometries simply change the length and thus effectively rescale *d*. Differences in the shape, and thus area per unit length, for the *h*- and *p*-type interfaces may have an effect, but we expect these differences to be small.

It should be noted that the radius of the unperturbed HD, a=0, including finite separation correction, is

(Equation 8) $$R_{h0}\left(d\right)=R_{h}{|}_{a=0}\;=\left(\pi -2\right)d+\sqrt{{\left(\pi -2\right)}^{2}d^{2}+\frac{2A_{sys}-A_{m}}{\pi }}\text{.}$$

This can be thought of as a finite-*d* correction to the previously used $R_{h0}=R_{h}{|}_{a=0,d=0}=\sqrt{\left(2A_{sys}-A_{m}\right)/\pi }$. The corrected version, $R_{h0}\left(d\right)$, which we denote using the *d* dependence, and the corresponding area, $A_{h0}\left(d\right)=\pi R_{h0}^{2}\left(d\right)$, are more useful when comparing with experiments.

## Appendix B: Self-consistent field theory implementation

Using numerical self-consistent field theory (SCFT) (56,57), we determine stable and metastable lipid configurations, such as planar membranes and HDs. We also use the string method (41,42) to find transition paths, and thus locate free-energy barriers between states. Our implementation of SCFT, summarized below, closely resembles that of (67) and utilizes a modified version of the same simulation program.

### Equilibrium SCFT

The lipids are approximated as linear AB diblock copolymers with *N* segments. The majority block, with a molecular volume fraction *f*, represents the tail; and the minority block, with volume fraction 1−f, represents the head. Water is represented by a short homopolymer, with $N_{s}=N/10$ segments. The strength of interactions between unlike monomers is controlled by the Flory-Huggins parameter, χN. We set the head-water interaction energy to 0, meaning that head monomers have no preference between water versus other head monomers, and we typically use χN=30 for the head-tail and water-tail interactions.

In SCFT, nonbonded interactions are represented by fields, $w_{\gamma }\left(r\right)$, which act on γ-type monomers. In our case, γ=A for the tail groups, γ=B for the head and water. The lipids are approximated as Gaussian chains, allowing us to calculate the statistics of the lipids and thus the probability distribution and concentration of each point on the chain, i.e., monomer. This is done through the use of “propagators,” which represent the partition function of a portion of the molecule. If the lipid is represented as a Gaussian chain, the “forward” propagator satisfies

(Equation 9) $$\frac{\partial q\left(r,s\right)}{\partial s}=\left[\frac{R_{0}}{6}\nabla^{2}-w\left(r,s\right)\right]q\left(r,s\right)$$

and represents the partition function for the portion of the molecule from 0 to *s*, given that monomer *s* is fixed at spatial position r. *s* is the normalized position along the molecule and $R_{0}$ is the equilibrium end-to-end distance for a noninteracting molecule. To map SCFT distances onto real systems we match the bilayer width, $D\approx 1.2R_{0}\approx 4\;\text{nm}$, found in theory and experiments (68). $w\left(r,s\right)=w_{A}\left(r\right)$ for *A* monomers and $w_{B}\left(r\right)$ for *B* monomers. We find the propagator by numerically solving this equation starting from uniform initial conditions. The “back” propagator, $q^{†}\left(r,s\right)$, is found similarly, but with one side of Eq. 9 multiplied by −1.

Monomer concentrations are given by

(Equation 10) $$\varphi \left(r,s\right)=\frac{V}{Q}q\left(r,s\right)q^{†}\left(r,s\right)$$

where

(Equation 11) $$Q=\int drq\left(r,s\right)q^{†}\left(r,s\right)$$

is the partition function for the molecule in the fields. The concentration of each monomer type is calculated by integrating φ(r,s) over appropriate ranges of *s*, i.e., s=0 to *f* for *A* and s=f to 1 for *B*. A similar approach is used for the solvent, represented by a shorter homopolymer feeling only the *B* field.

Since the fields represent nonbonded interactions and are thus related to the monomer concentrations, $\varphi_{\gamma }$, we know that the fields produced by some concentration profile are given by

$$w_{A}\left(r\right)=\chi N\varphi_{B}\left(r\right)+\xi \left(r\right)$$

(Equation 12) $$w_{B}\left(r\right)=\chi N\varphi_{A}\left(r\right)+\xi \left(r\right)$$

where ξ(r) is a pressure field, which ensures incompressibility, $\varphi_{A}+\varphi_{B}=1$. We adjust the fields until these equations are satisfied, i.e., until the fields produced by the concentration profile are the same as the field that produced the concentration profile. These solutions are “self-consistent” and correspond to stable or metastable configurations. The canonical free energy of the resulting configuration is given by

(Equation 13) $$\frac{F}{\sqrt{\bar{N}}k_{B}T}=-{\bar{\varphi }}_{l}\;\ln\;Q_{l}-\frac{N{\bar{\varphi }}_{s}}{N_{s}}\ln\;Q_{s}\;+\int \left[\chi N\varphi_{A}\left(r\right)\varphi_{B}\left(r\right)-w_{A}\left(r\right)\varphi_{A}\left(r\right)-w_{B}\left(r\right)\varphi_{B}\left(r\right)\right]dr$$

where $\bar{N}$ is the invariant polymerization index. $\bar{N}$ represents the degree of overlap of the lipids and is used as a fitting parameter determined by matching the bending modulus (see below), κ, of the lipids to our SCFT predictions. ${\bar{\varphi }}_{l}$ and ${\bar{\varphi }}_{s}$ are the average lipid and solvent concentrations, and satisfy ${\bar{\varphi }}_{l}+{\bar{\varphi }}_{s}=1$. $Q_{l}$ is the partition function for the lipids, calculated as above, and similarly for $Q_{s}$. The interaction energy is the sum of pair-wise interactions between monomers but, since these interactions are implemented through external fields acting on noninteracting chains, the interactions are double-counted. The integral term is the negative of the interaction energy and corrects for the double counting. SCFT has been applied to a variety of lipid and polymer problems (24,43,44,45,62,114,115). More detailed and rigorous derivations of SCFT are presented in (56,57).

Our HD calculations typically use system sizes of size $\left(32\times 32\times 8\right)R_{0}^{3}\approx \left(107\times 107\times 27\right)$ nm^3^. This is represented on a 400×400×100 grid. The relevant HD portion is cropped out of this large system. We solve Eq. 9 using a pseudospectral method (116), with a step size along the lipid of Δs=1/60, i.e., discretize the lipid into 60 steps.

To compare with experiments or particle-based simulations it is useful to calculate bulk membrane properties. Since SCFT gives us direct access to the free energy, we can easily determine the tension by calculating the grand canonical free energy per unit area of a planar membrane. The bending modulus can be calculated by fitting the free energy of a cylindrical membrane to the usual form (117) and for f=0.8 and χN=30 we obtain $\kappa =0.209\sqrt{\bar{N}}k_{B}T$. We use a similar approach to calculate the spontaneous monolayer curvature, $c_{0}$, but using a reflecting boundary so that we can consider only a single monolayer. Fitting the free energy versus radius gives us $c_{0}$ for a monolayer with the headgroups pointing “inward,” which is the negative of the $c_{0}$ for headgroups pointing “outward.” When reporting data we adopt the outward convention to match the literature (69,70).

To calculate line tensions in SCFT, we use systems similar to those in Fig. 2, but duplicated to use periodic boundary conditions. Example systems are shown in Fig. 14. The location of the interface (edge, threefold junction or rim pore) adjusts in position such that the membrane is tensionless. The line tension is thus given by a spatial derivative of the free energy at fixed volume, $\lambda =\frac{1}{2}\frac{dF}{dL}{|}_{V}$, where *L* is the dimension along the junction. For f=0.8 and χN=30, $\lambda_{e}=0.288\sqrt{\bar{N}}k_{B}T/R_{0}\approx 8.27k_{B}T/\text{nm}$, $\lambda_{h}=0.290\sqrt{\bar{N}}k_{B}T/R_{0}\approx 8.233k_{B}T/\text{nm}$, and $\lambda_{p}=0.160\sqrt{\bar{N}}k_{B}T/R_{0}\approx 4.59k_{B}T/\text{nm}$. $\lambda_{p}$ and $\lambda_{h}$ depend on the spacing between the apposing bilayers, and for the above values we chose a typical spacing for an HD of $2d=3.2R_{0}$. The value for $\lambda_{p}$ is slightly lower than that expected from the bending energy, $\pi \kappa /2d=0.205\sqrt{\bar{N}}k_{B}T/R_{0}$. The *p*-type interface is not a perfect semicircle, but bulges outward, distributing the curvature and lowering the overall bending energy. Absolute (nonscaled) line tensions over the range of interest are shown in Fig. 15. Membrane monolayer spontaneous curvature, $c_{0}$, and bending energy, $\kappa_{m}$, are shown in Fig. 16.

### Incorporating proteins and restricting flip-flops

The majority of our SCFT calculations are conducted as described above. Due to the nature of SCFT, lipids may move entirely freely, and we do not capture, for example, behavior that emerges from slow lipid diffusion. In real lipid membranes, lipids do not diffuse so freely. A restriction on lipid flip-flops may affect membrane dynamics, as is discussed in more detail later. We can incorporate this into SCFT by preventing the leaflets on either side of a membrane from mixing. This is done by treating the lipids on either side as different molecular species, with fixed lipid numbers, and adding a repulsive interaction between the headgroups of these species. The procedure is otherwise similar to above, with an extra field and molecular species. The results are not sensitive to the strength of the repulsion, as long as it is strong enough for the different headgroup phase segregate.

External influences, such as proteins, can be incorporated through the use of external fields, representing interactions with the lipids and solvent. The field equations are modified to incorporate the external fields, ${\tilde{w}}_{A}\left(r\right)$, ${\tilde{w}}_{\text{head}}\left(r\right)$, and ${\tilde{w}}_{\text{water}}\left(r\right)$, resulting in the field conditions

(Equation 14) $$w_{A}\left(r\right)=\chi N\varphi_{B}\left(r\right)+\xi \left(r\right)+{\tilde{w}}_{A}\left(r\right)$$

(Equation 15) $$w_{\text{head}}\left(r\right)=\chi N\varphi_{A}\left(r\right)+\xi \left(r\right)+{\tilde{w}}_{\text{head}}\left(r\right)$$

(Equation 16) $$w_{\text{water}}\left(r\right)=\chi N\phi_{A}\left(r\right)+\xi \left(r\right)+{\tilde{w}}_{\text{water}}\left(r\right)$$

where now, rather than having a single $w_{B}$, it is useful to have separate fields acting on the head and water. If the external fields are absent, these fields are the same and equal to $w_{B}\left(r\right)$ discussed above. The free energy is similarly modified,

(Equation 17) $$\frac{F}{\sqrt{\bar{N}}k_{B}T}=-{\bar{\varphi }}_{l}\;\ln\;Q_{l}-{\bar{\varphi }}_{s}\;\ln\;Q_{s}+\int dr\left[\chi N\varphi_{A}\left(r\right)\varphi_{B}\left(r\right)-w_{A}\left(r\right)\varphi_{A}\left(r\right)-w_{B}\left(r\right)\varphi_{B}\left(r\right)+{\tilde{w}}_{A}\left(r\right)\varphi_{A}\left(r\right)+{\tilde{w}}_{\text{head}}\left(r\right)\varphi_{\text{head}}\left(r\right)+{\tilde{w}}_{\text{water}}\left(r\right)\varphi_{\text{water}}\left(r\right)\right]$$

As described above, the purpose of the integral term is to subtract the double-counted interaction energy, but the interactions with the externally imposed fields ($\tilde{w}$ s) are not double-counted. We must therefore subtract their contribution from the integral. This is equivalent to using the *w*s in Eqs. 14, 15, and 16 without the $\tilde{w}$ terms.

We use this technique to mimic the effect of proteins that wrap around an HD, keeping its size constant. As a simple ansatz, we set a toroidal region that excludes lipids. Placing the torus in the x−y plane and controlling its geometry using parameters labeled *T*,

(Equation 18) $${\tilde{w}}_{T}\left(r,T_{R},T_{w},{\tilde{w}}_{0}\right)=w_{0}\;e^{-\frac{{\left(\sqrt{x^{2}+y^{2}}-T_{R}\right)}^{2}}{2T_{w}^{2}}-\frac{z^{2}}{2T_{w}^{2}}}$$

(Equation 19) $${\tilde{w}}_{A}\left(r\right)={\tilde{w}}_{T}(r,T_{R},T_{w},{\tilde{w}}_{0}$$

(Equation 20) $${\tilde{w}}_{\text{head}}\left(r\right)={\tilde{w}}_{T}\left(r,T_{R},T_{w},{\tilde{w}}_{0}\right)$$

(Equation 21) $${\tilde{w}}_{\text{water}}\left(r\right)=0\text{.}$$

We typically use a thin torus, of width $T_{w}=0.25R_{0}$, and an amplitude $w_{0}=2\chi N$. The radius, $T_{R}$, will vary. The results presented are not sensitive to the particular geometry of the torus, with the exception of the influence of the radius, $T_{R}$, on the HD radius, as will be discussed. It may seem strange to exclude only the lipids from the region occupied by the protein, as opposed to the lipids and water; however, the latter makes the calculation more cumbersome and the outcome is equivalent: a region where no water or lipid is allowed versus a region where only water is allowed has an equivalent effect on membrane conformations.

### The string method

In addition to stable and metastable structures, we are also interested in the mechanisms by which these structures transform into one another. Specifically, we are interested in the mechanisms of pore formation and growth. Finding the optimal paths between stable or metastable configurations is done using the string method (41,42,118,119,120,121,122) to find the MFEP, connecting configurations (41,42). The MFEP provides a thermodynamically reversible estimate for the most probable pathway between metastable states.

The metastable points (at the ends of the MFEP) satisfy the field equations above, Eq. 12, or the modifications thereto; however, the intermediate points are not metastable and have a residual chemical potentials

$$\Delta w_{A}\left(r\right)=\chi N\varphi_{B}\left(r\right)+\xi \left(r\right)-w_{A}\left(r\right)$$

(Equation 22) $$\Delta w_{B}\left(r\right)=\chi N\varphi_{A}\left(r\right)+\xi \left(r\right)-w_{B}\left(r\right)$$

which are functional derivatives of the free energy with respect to the concentration, i.e., free energy gradients in configuration space. They therefore “point” in the “direction” in configuration space that decreases the free energy. The MFEP corresponds to a path that is locally optimal, which entails that the residual chemical potentials are purely “parallel” to the string.

To enforce incompressibility over the entire MFEP, it is convenient to write the fields in terms of a composition field, $w_{-}\left(r\right)=\left(w_{A}\left(r\right)-w_{B}\left(r\right)\right)/2$ and a pressure field $w_{+}\left(r\right)=\left(w_{A}\left(r\right)+w_{B}\left(r\right)\right)/2$, which couple to $\varphi_{-}\left(r\right)=\varphi_{A}\left(r\right)-\varphi_{B}\left(r\right)$ and $\varphi_{+}\left(r\right)=\varphi_{A}\left(r\right)+\varphi_{B}\left(r\right)$, respectively. The residual chemical potentials then become

(Equation 23) $$\Delta w_{-}\left(r\right)=\chi N\varphi_{-}\left(r\right)-2w_{-}\left(r\right)$$

(Equation 24) $$\Delta w_{+}\left(r\right)=\chi N\left(\varphi_{+}\left(r\right)-1\right)\text{.}$$

This allows us to solve for $\Delta w_{+}\left(\vec{r}\right)=0$ along the entire MFEP even though $\Delta w_{-}\left(\vec{r}\right)$ is finite throughout the transformation.

To calculate the MFEP, the string method involves considering *m* configurations indexed by *i* in the range 1≤i≤m. This is a discretization along the reaction coordinate, α, into *m* points. We label the fields $w_{-}^{\left(i\right)}$ and $w_{+}^{\left(i\right)}$, and concentrations $\varphi_{-}^{\left(i\right)}$ and $\varphi_{+}^{\left(i\right)}$. α is determined by calculating the Euclidean distance between adjacent points on the string and normalizing the total string length to 1, i.e.,

(Equation 25) $$\alpha_{i+1}-\alpha_{i}=\frac{\int {\left[w_{-}^{\left(i+1\right)}\left(r\right)-w_{-}^{\left(i\right)}\left(r\right)\right]}^{2}dr}{\sum_{j=1}^{m-1}\int {\left[w_{-}^{\left(j+1\right)}\left(r\right)-w_{-}^{\left(j\right)}\left(r\right)\right]}^{2}dr}\text{.}$$

The ends of the string, i=1 and i=m, are updated as described previously, and relax into stable or metastable configurations. The intermediate points are updated similarly but only the portion of Eq. 23 perpendicular to the string is used (this is clarified below) and the configurations are shifted along the string, using a cubic interpolation, so that they remain equidistant in α, i.e., enforcing $\alpha_{i}=\left(i-1\right)/\left(m-1\right)$ (42).

The perpendicular component of Eq. 23 is calculated by subtracting the component that is parallel to the string,

(Equation 26) $$\Delta w_{-}^{⊥}\left(r,\alpha \right)=\Delta w_{-}\left(r,\alpha \right)-\Delta w_{-}^{∥}\left(r,\alpha \right)$$

where the field, $w_{-}\left(r\right)$, which is represented on a discrete grid of *M* points, is treated as an *M*-dimensional vector and “perpendicular” and parallel are defined in this *M*-dimensional space, as is the dot product below. The parallel component is

(Equation 27) $$\Delta w_{-}^{∥}\left(r,\alpha \right)=\hat{\dot{w}}\left(r,\alpha \right)\left(\Delta w_{-}\left(r,\alpha \right)\cdot \hat{\dot{w}}\left(r,\alpha \right)\right)$$

where

(Equation 28) $$\hat{\dot{w}}\left(r,\alpha \right)=\frac{dw_{-}\left(r,\alpha \right)}{d\alpha }{\left|\frac{dw_{-}\left(r,\alpha \right)}{d\alpha }\right|}^{-1}$$

is the unit vector pointing parallel to the string. The term in parentheses in Eq. 27 is the magnitude of the component of $\Delta w_{-}\left(r,\alpha \right)$ pointing parallel to the string.

In addition to making the update more direct, as it is only acting perpendicular to the string, this modification allows the update to more accurately monitor the degree of convergence. The completely converged string satisfies $\Delta w_{-}^{⊥}\left(r,\alpha \right)=0$; however, due to the computationally intensive nature of these calculations, we are typically satisfied with a root mean-squared error of $\underset{˜}{<}10^{-3}$.

## Appendix C: Molecular dynamics simulation and techniques

GROMACS was utilized to conduct all molecular dynamics simulations (71). We use the leap-frog algorithm with a time step of 20 fs to integrate Newton’s equations of motion. The Berendsen thermostat maintained the temperature at 300 K. To ensure the continuity of forces and their derivatives at the cutoff radius, the nonbonded and electrostatic interactions were shifted to zero via the GROMACS shift function in the range 0.9–1.2 nm and 0.0–1.2 nm, respectively. Periodic boundary conditions were applied in all three Cartesian dimensions.

We created an HD in a two apposing bilayer system by applying an external field (123) and subjecting the membrane to tension. This procedure resulted in a fast formation of an HD. The initial hydrophobic bridge formed in only a few ns, but expanded into an HD over approximately 100 ns. This procedure did not result in an equal area per lipid in the three disconnected monolayers, thus further equilibration steps were required.

Since spontaneous flip-flops of lipids between monolayers is an extremely rear event, we promote equilibration of the monolayers by adding small pores of radius 1−2 nm far away from the HD in each of the apposing membranes, which were kept open by means of a potential on lipid tails (124). After ≈1μs of equilibration, the area per lipid, σ, in the HD and the two apposing membranes was 0.593±0.005 and 0.595±0.003 nm^2^, respectively. Note that this area per lipid is larger than that of a tensionless membrane, $\sigma_{0}=0.5787\pm 0.0003$ nm^2^, reflecting the presence of an HD. Thereafter, properties were analyzed during a 200 ns production run.

To calculate the line tensions, $\lambda_{e}$, $\lambda_{p}$, and $\lambda_{h}$, we used the three system configurations depicted in Fig. 17, where the edges of a membrane, a fusion pore, and a HD align along the *x* axis. The double-layer systems are essentially dehydrated tubes, with the amount of water adjusted to set the spacing, *d*, and this spacing is small but large enough that the hydration repulsion can be neglected (124). In the NVT ensemble, we estimated the line tension, λ, at vanishing membrane tension, from the pressure anisotropy (72),

(Equation 29) $$\lambda =\frac{L_{y}L_{z}}{2}\langle \frac{P_{yy}+P_{zz}}{2}-P_{xx}\rangle \text{,}$$

where $L_{y}$ and $L_{z}$ denote the box lengths along the *y* and *z* axes, and $P_{xx}$, $P_{yy}$, and $P_{zz}$ are the components of the pressure tensor. The factor 1/2 arises because the system with periodic boundaries contains two edges. The system parameters used for line tension calculations and the following verification for DMPC lipids, as well as the systems used to study stable and critical pores, are summarized in Table 1. Similar parameters were used for simulating POPC systems for line tension calculations. Line tensions found for POPC and DMPC are given in Fig. 15.

To verify equilibration, we validated the properties of the HD and containing system against the Laplace pressure relationship, $\Sigma =\lambda_{h}/R_{h}$. The system containing an HD of radius $R_{h}=\left(9.2\pm 2.6\right)\;\text{nm}$ and line tension $\lambda_{h}=\left(11\pm 2\right)k_{B}T/\text{nm}$ has a measured membrane tension of $\Sigma =\left(1.48\pm 0.50\right)k_{B}T/{\text{nm}}^{2}$, which is consistent with the relation above.

Line tensions of domain boundaries in lipid-separating membranes are typically on the order of picoNewtons (125,126). Edge tensions, $\lambda_{e}$, are typically larger, on the order of several tens of picoNewtons, for example, for DOPC (both tails unsaturated) experiments found $\lambda_{e}\approx 28\pm 3$ pN (87). MARTINI simulations obtained a larger value, 65±3 pN, for the same lipid type (127), which is similar to our POPC value, $14.5\pm 0.5k_{B}T/\text{nm}\approx 59.6\pm 2$ pN as well as the POPC edge tension found from atomistic simulations, 57±10 pN (72). Our shorter-tailed lipid, DMPC, has a smaller edge tension, $9.4\pm 0.5k_{B}T/\text{nm}\approx 38.6\pm 2$ pN, in agreement with atomistic simulations of membrane edges (72).

Although our edge tension for POPC matched those found by previous MARTINI simulations of DOPC (127), our finding for the threefold junction is significantly higher, at $\lambda_{h}=11.5\pm 1.0k_{B}T/$ nm ≈47.3±4 pN, compared to Risselada’s finding of 23±10 pN. Their calculation considered a junction of three planar membranes, intersecting at $120^{\circ }$, whereas our HDs deviate from this ideal angle, and have additional membrane curvature. The difference may also be partially due to differences between POPC and DOPC.

The line tension of the fusion pore, $\lambda_{p}$, can be estimated from the elastic bending energy using πκ/2d, where κ is the bending rigidity and 2d is the distance between the centers of mass of the two apposing membranes. Using known bending rigidities of MARTINI POPC and DMPC membranes, $\kappa_{POPC}=21.2\pm 1.2$ and $\kappa_{DMPC}=22.7\pm 0.7$ k_*B*_T (128,55), and $2d_{POPC}=4.7\pm 0.1$ nm and $2d_{DMPC}=4.3\pm 0.1$ nm we obtain line tensions in the range of 60–70 pN, which is close to our calculated line tensions of $\lambda_{p}=17.5\pm 1.3k_{B}T\approx 71.9\pm 5$ pN and $18.3\pm 1.5k_{B}T\approx 75.2\pm 6$ pN for DMPC and POPC membranes, respectively.

## Supporting material

Video S1. Fluctuating pore in hemifusion diaphragm
